# High PPT1 expression predicts poor clinical outcome and PPT1 inhibitor DC661 enhances sorafenib sensitivity in hepatocellular carcinoma

**DOI:** 10.1186/s12935-022-02508-y

**Published:** 2022-03-11

**Authors:** Jianjun Xu, Zhe Su, Xiang Cheng, Shaobo Hu, Wenjie Wang, Tianhao Zou, Xing Zhou, Zifang Song, Yun Xia, Yang Gao, Qichang Zheng

**Affiliations:** 1 Department of Hepatobiliary Surgery, Union Hospital, Tongji Medical College, Huazhong University of Science and Technology, No.1277, Liberation Avenue, Jianghan District, Wuhan, 430022 China; 2 Cancer Center, Union Hospital, Tongji Medical College, Huazhong University of Science and Technology, Wuhan, 430022 China; 3 Department of General Surgery, Tongji Hospital, Tongji Medical College, Huazhong University of Science and Technology, Wuhan, 430030 China

**Keywords:** Hepatocellular carcinoma, PPT1, Autophagy, Lysosome, Targeted therapy, Immune infiltration, Prognosis

## Abstract

**Background:**

Adaptive resistance and side effects of sorafenib treatment result in unsatisfied survival of patients with hepatocellular carcinoma (HCC). Palmitoyl-protein thioesterase 1 (PPT1) plays a critical role in progression of various cancers. However, its role on prognosis and immune infiltrates in HCC remains unclarified.

**Methods:**

By data mining in the Cancer Genome Atlas databases, the role of PPT1 in HCC were initially investigated. Furthermore, HCC cell lines Hep 3B and Hep 1-6 were treated with DC661 or siRNA against PPT1. The biological function of PPT1 was determined by CCK-8 test, colony formation assay, TUNEL staining, immunofluorescence staining, Western blot test, and PI-Annexin V apoptosis assays *in vitro*. Animal models of subcutaneous injection were applied to investigate the therapeutic role of targeting PPT1.

**Results:**

We found that PPT1 levels were significantly upregulated in HCC tissues compared with normal tissues and were significantly associated with a poor prognosis. Multivariate analysis further confirmed that high expression of PPT1 was an independent risk factor for poor overall survival of HCC patients. We initially found that PPT1 was significantly upregulated in sorafenib-resistant cell lines established in this study. Upon sorafenib treatment, HCC cells acquired adaptive resistance by inducing autophagy. We found that DC661, a selective and potent small-molecule PPT1-inhibitor, induced lysosomal membrane permeability, caused lysosomal deacidification, inhibited autophagy and enhanced sorafenib sensitivity in HCC cells. Interestingly, this sensitization effect was also mediated by the induction mitochondrial pathway apoptosis. In addition, the expression level of PPT1 was associated with the immune infiltration in the HCC tumor microenvironment, and PPT1 inhibitor DC661 significantly enhanced the anti-tumor immune response by promoting dendritic cell maturation and further promoting CD8^+^ T cell activation. Moreover, DC661 combined with sorafenib was also very effective at treating tumor models in immunized mice.

**Conclusions:**

Our findings suggest that targeting PPT1 with DC661 in combination with sorafenib might be a novel and effective alternative therapeutic strategy for HCC.

**Supplementary Information:**

The online version contains supplementary material available at 10.1186/s12935-022-02508-y.

## Introduction

Liver cancer (hepatocellular carcinoma, HCC) is one of the most deadly diseases of man; it is the seventh most commonly diagnosed cancer, the third leading cause of cancer deaths worldwide, and a serious threat to people’s lives and health [1]. With the development of molecular-targeted therapies, HCC therapy has entered a new era, in which sorafenib is improving the survival of patients with advanced HCC [2]. Sorafenib, a multi-kinase inhibitor, blocks tumor cell proliferation by specifically targeting multiple growth factor pathways and plays a role in anti-angiogenesis. Two large phase-III randomized clinical trials, including the SHARP trial, have demonstrated the survival benefits of sorafenib in patients with advanced HCC [3, 4]. However, the survival benefit seen in the sorafenib treatment group was merely modest. In a large trial conducted in Asia, the median survival of those given sorafenib was only 2.3 months longer than that of the placebo group [4]. Part of this unsatisfactory response may be due to sorafenib resistance [4, 5], and the side effects also discourage use of the drug. In order to extend survival in HCC patients, combination therapies targeting the underlying resistance mechanisms may be better treatment options, as they have the potential to circumvent resistance and increase tumor cell sensitivity to sorafenib. Therefore, a more detailed understanding of the main mechanisms of sorafenib resistance may help to improve its therapeutic effects on HCC.

Although sorafenib appears to be effective in extending median survival in HCC patients and to have limited side effects, it may induce resistance in many patients [6], which presents a barrier to prolonging their overall survival. Due to the genetic heterogeneity of HCC, some HCC cells and patients are initially resistant to sorafenib, i.e., they show what is referred to as primary resistance [7]. The exact mechanism for this resistance, however, remains unclear. Acquired resistance to sorafenib, which is also of great concern, involves multiple mechanisms, such as autophagy, the PI3K/Akt and JAK-STAT pathways, the activation of hypoxia-induced pathways, and epithelial-mesenchymal transformation. Autophagy is a self-protection mechanism of the body in response to various stress-induced signals, and its involvement in HCC has achieved general consensus [7]. Activation of mild autophagy can promote the survival of HCC cells in the absence of nutrients, whereas excessive autophagy can promote the apoptosis of tumor cells. Sorafenib was originally developed as a Raf kinase inhibitor; however, it also inhibits other tyrosine kinases, such as VEGR-2, Flt-3, and c-Kit. Studies have shown that sorafenib can inhibit the mammalian target of rapamycin (mTOR) signaling pathway, a major regulatory pathway of autophagy, promoting mild autophagy and, thus, HCC cell survival and limiting the efficiency of sorafenib [8]. Therefore, to prolong the survival of HCC patients, combining autophagy inhibition with sorafenib administration could be a potentially valuable strategy for reversing adaptive drug resistance and increasing the sensitivity of HCC cells to sorafenib.

Because both normative and non-normative autophagies depend on lysosomal degradation, lysosomes provide ideal targets for autophagy inhibition [9, 10]. Preclinical studies showing that targeting lysosomes can improve anticancer therapies have led to more than 20 phase I/II clinical trials combining anticancer agents with the lysosomal inhibitor hydroxychloroquine (HCQ) [11]. Although targeting lysosomes can produce measurable autophagy inhibition, the inhibition of autophagy in patient tumors by HCQ is not consistent [12, 13]. Therefore, more effective lysosomal inhibitors are urgently needed. Palmitoyl-protein thioesterase 1 (PPT1) is known to be widely and significantly overexpressed in a variety of cancers, including breast, thyroid, and gastric cancers [14]. Higher expression levels of PPT1 in tumors are associated with shorter overall survival for a variety of cancers, including head and neck, esophageal, and renal cell cancers [14]. Recently, a selective and potent small-molecule PPT1 inhibitor, DC661, has been formulated [14]. Studies have shown that DC661 has the strongest lysosomal-inhibition compared with other monomer or dimer chloroquine (CQ) derivatives [14], suggesting that the targeting of PPT1 expression in combination with sorafenib treatment for HCC is a potentially valuable therapeutic strategy. However, the potential mechanism by which the DC661 affects lysosomal function remains unclear.

To date, there have been no reports of whether DC661 reverses sorafenib adaptive resistance or increases the sorafenib sensitivity of HCC. In this study, we attempted to test these hypotheses with in vitro HCC cells and in vivo HCC tumor models. Our results suggest that the inhibition of PPT1 leads to highly permeable lysosomal membranes by impairing the heat shock protein-70.1 (HSP70.1)/bis(monoacylglycero)phosphate (BMP)/acidic sphingomyelinase (ASM) pathway, inhibits autophagy, induces apoptosis via the mitochondrial pathway, and thus is a potential strategy for overcoming sorafenib resistance. Combining sorafenib with the selective PPT1 inhibitor DC661 may be a novel therapeutic strategy against HCC.

## Results and discussion

### Role of PPT1 in HCC

**Fig. 1:**
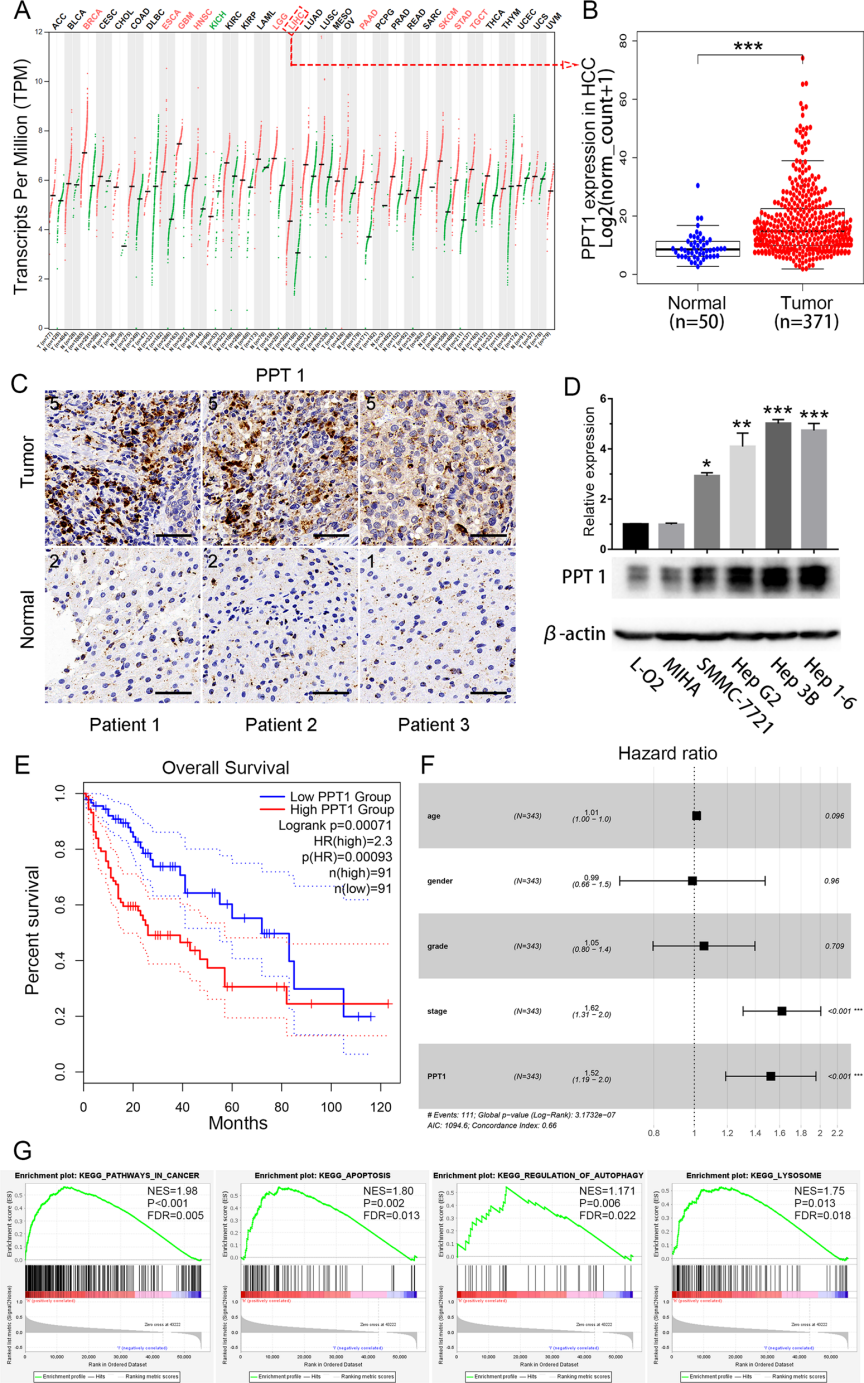
Role of PPT1 in HCC. **A** PPT1 mRNA expression in various normal human tissues and tumor tissues. **B** TCGA database-based comparison of PPT1 mRNA expression in HCC tissues (n = 371) and normal liver tissues (n = 50); ***P < 0.001. **C** Representative immunohistochemical results of PPT1 in patient-derived HCC tissue and normal liver tissue. Scale bar, 50 μm. **D** Expression of PPT1 in a panel of normal liver cell lines (L-O2 and MIHA) and HCC cell lines. On Western blot analysis, PPT1 was found to be preferentially expressed in HCC cell lines, Hep 3B and Hep 1-6. **E** Kaplan–Meier curves of overall survival (OS). High PPT1 expression was correlated with poor OS in HCC patients. **F** Cox proportional hazards regression model analysis of OS. **G** Gene set enrichment analysis (GSEA) using the TCGA dataset. PPT1 overexpression was significantly correlated with “KEGG_PATHWAYS_IN_CANCER”, “KEGG_APOPTOSIS”, “KEGG_REGULATION_OF_AUTOPHAGY”, and “KEGG_LYSOSOME” pathways. NES: normalized enrichmentscore

We found that PPT1 expression was elevated in HCC tissue. Using the mRNA-seq data available in the Cancer Genome Atlas (TCGA) database, we reviewed PPT1 expression in normal human tissues. Compared with various normal tissues, a variety of tumor tissues showed high levels of PPT1 expression (Fig. 1A). When compared with other normal tissues, normal liver tissue showed low basal PPT1 expression at the mRNA level (Fig. 1A). However, PPT1 mRNA expression in HCC tumor tissue was significantly higher than that in normal liver tissue (Fig. 1B). Consistent with this, we confirmed there were different PPT1 mRNA expression levels between HCC tissue and paired normal tissue (Additional file 1: Fig. S1). In addition, immunohistochemical analysis confirmed that PPT1 protein expression in the HCC tissue was significantly higher than that in normal liver tissue (Fig. 1C, Additional file 1: Fig. S2). Most of the HCC cell lines also showed higher PPT1 protein expression than the normal liver cells (L-O2 and MIHA) in vitro (Fig. 1D).

High PPT1 expression independently predicted poor progression-free survival (PFS) and overall survival (OS) in HCC patients. A total of 364 HCC patients with complete PPT1 mRNA-seq data and clinical data in TCGA-LIHC (liver hepatocellular carcinoma) were enrolled to explore the clinical significance of PPT1. Kaplan–Meier analyses indicated that high PPT1 expression was generally associated with poor PFS (Fig. S3) and OS (Fig. 1E). We subsequently used a Cox regression multivariate analysis to confirm that high PPT1 expression was an independent risk factor of OS in HCC patients (Fig. 1F).

Gene set enrichment analysis (GSEA) was conducted to search for cancer-related pathways enriched in samples with high PPT1 expression. Four gene sets, “KEGG_PATHWAYS_IN_CANCER”, “KEGG_APOPTOSIS”, “KEGG_REGULATION_OF_AUTOPHAGY”, “KEGG_LYSOSOME”, were significantly enriched (Fig. 1G).

### Sorafenib induced autophagy and upregulated the expression of PPT1 in sorafenib-resistant HCC cells

**Fig. 2:**
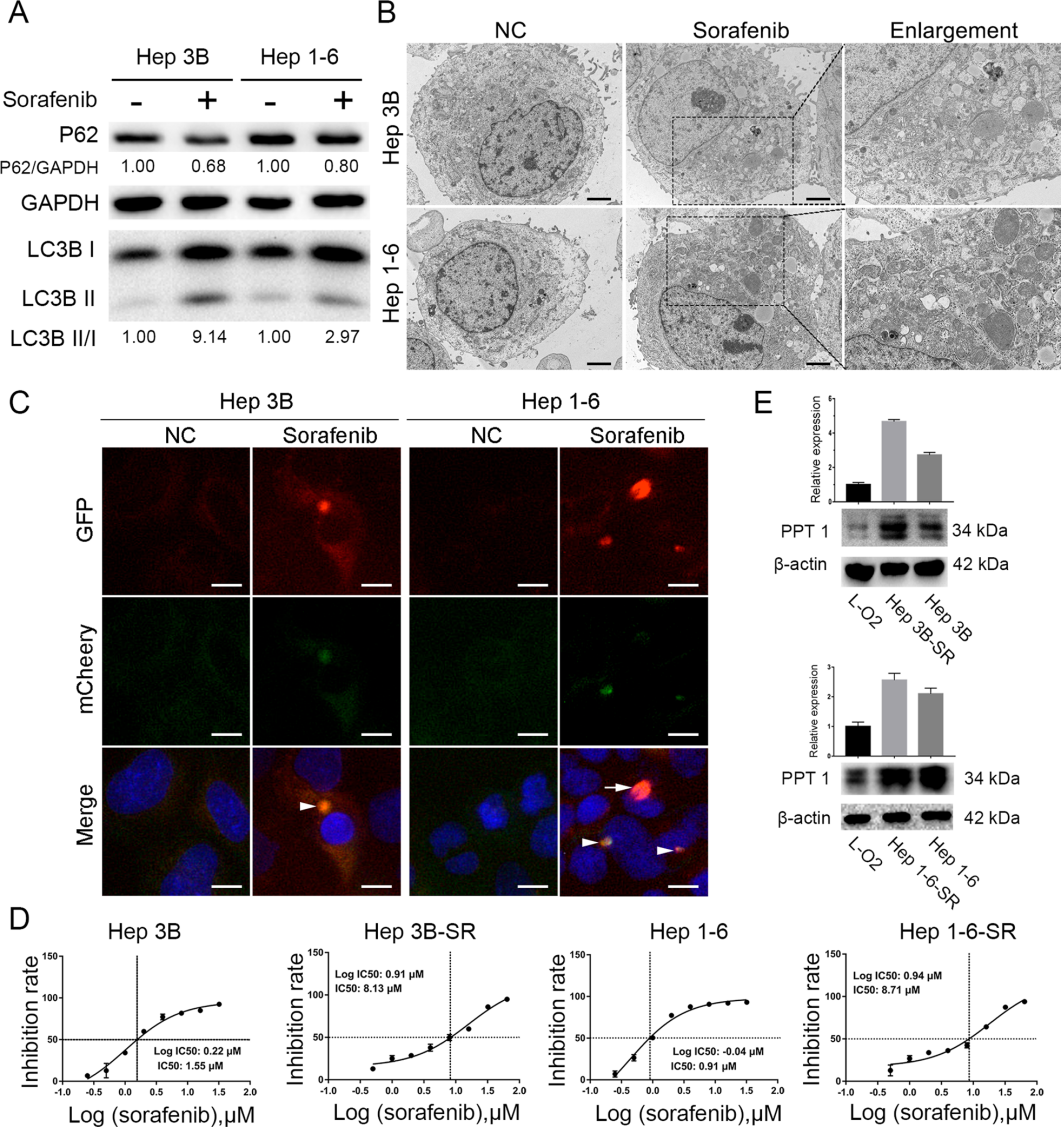
Sorafenib induced autophagy and upregulated the expression of PPT1 in sorafenib-resistant HCC cells. **A** Western blot showing increase in LC3-II in HCC cells after treatment with sorafenib (10 µΜ, 24 h). **B** Photographs from transmission electron microscopy showing an increase in autophagosomes in HCC cells after treatment with sorafenib (10 µΜ, 24 h). Scale bar, 2 μm. **C** Fluorescence microscopic images showing punctate fluorescence from transfected mCherry-GFP-LC3 constructs in HCC cells treated with sorafenib (10 µΜ, 24 h); nuclei are labeled with Hoechst 33,258. Arrowheads indicate typical examples of co-localized particles of GFP and mCherry signal, while the arrow points to a typical example of a particle with an mCherry signal but without GFP signal. Scale bar, 10 μm. **D** IC50 values of sorafenib (48 h) for Hep 3B, Hep 3B-SR, Hep 1**–**6, and Hep 1-6-SR cells determined by CCK-8 assay. The data shown are from three independent experiments. **E** Confirmation of the upregulation of PPT1 protein in sorafenib-resistant cells derived from Hep 3B and Hep 1**–**6 by Western blot analysis

The mTOR pathway is known to be a major regulatory pathway of autophagy, and negative regulation of the mTOR pathway promotes autophagy [15, 16]. Previous studies found that sorafenib inhibited the PI3Κ/AΚT/mTOR signaling pathway by inhibiting mammalian target of rapamycin complex 1 (mTORC1) activity and induced autophagy [8]. To examine the effect of sorafenib on human HCC-cell autophagy, we treated HCC cell lines Hep 3B and Hep 1-6 with sorafenib in vitro. First, we assessed the expression of LC3, which is considered to be the most critical signature protein in the autophagy signaling pathway, by Western blot test. The amount of LC3-II is proportional to the number of autophagosomes. After the fusion of an autophagosome and lysosome, LC3-II is degraded by lysosome proteases. When HCC cell lines were treated with 10 µM of sorafenib, the amounts of LC3-I and LC3-II increased significantly (Fig. 2A). The LC3-II/LC3-I ratio was higher in the sorafenib treatment group than the control group (Fig. 2A). In addition, transmission electron microscopy showed a large number of autophagic vacuoles, i.e., autophagosomes and possibly autolysosomes, in the sorafenib-treated HCC cells, but there were few vacuoles in the control cells (Fig. 2B).

To clarify whether the sorafenib-induced autophagosome accumulation was the result of inducing autophagosome formation or inhibiting autophagosome degradation, we first measured the amount of P62 by Western blot test. P62 is one of the marker proteins that reflect autophagy activity, and its content indirectly indicates the clearance level of autophagosomes. When Hep 3B and Hep 1**–**6 cells were treated with sorafenib, the amount of P62 was reduced despite the accumulation of LC3-II, suggesting that LC3-II accumulation was related to autophagy degradation. Our findings suggested that the LC3-II accumulation induced by sorafenib was due to the activation of autophagosome formation and not simply because autophagosome-degradation steps were inhibited. In addition, through the experiment of Ad-mCherry-GFP-LC3 adenovirus infection, we further confirmed that sorafenib could promote autophagy flow (Fig. 2C). From our findings and other evidence, we concluded that sorafenib could induce autophagy and cause adaptive drug resistance [8, 17, 18].

To further investigate the correlation between HCC-cell PPT1 expression and sorafenib response in vitro, we first determined the expression level of PPT1 in a group of HCC cell lines (Fig. 1D). We then measured the 50% inhibition concentration (IC50) values of these HCC cell lines after sorafenib treatment. Sorafenib-resistant clones of Hep 3B and Hep 1-6 cell lines were established by dose escalation at low concentration combined with intermittent shock at high dosage, and the IC50 values reached 8.13 µM (Hep 3B-SR) and 8.71 µM (Hep 1-6-SR) (Fig. 2D). The regulatory role of PPT1 in sorafenib resistance was further evidenced by the upregulation of PPT1 protein levels in our established sorafenib-resistant HCC cell lines, Hep 3B-SR and Hep 1-6-SR, in comparison to the controls (Fig. 2E).

### PPT1 inhibitor DC661 inhibited autophagy by inhibiting lysosomes

**Fig. 3:**
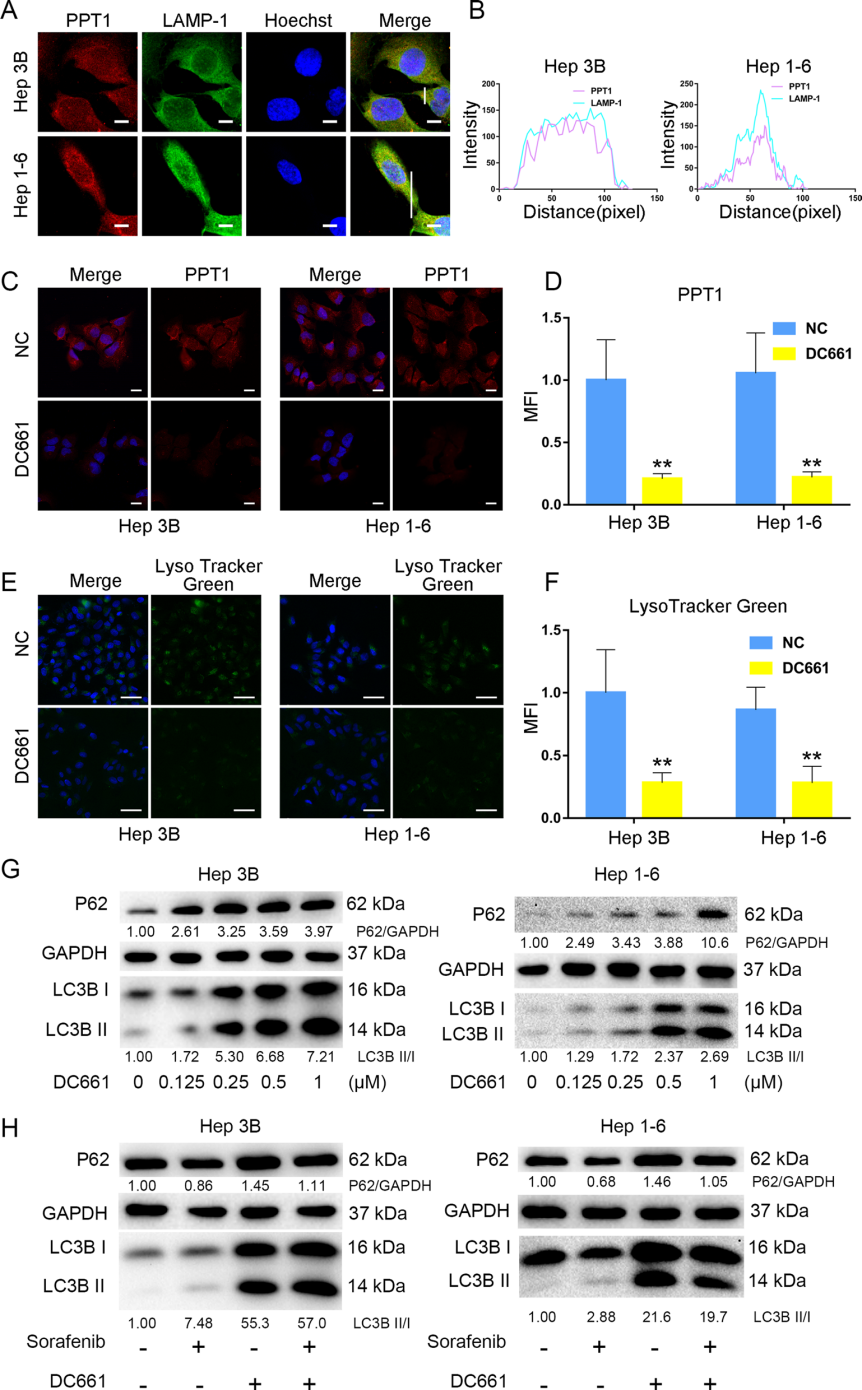
PPT1 inhibitor DC661 inhibited autophagy by inhibiting lysosomes. **A** Confocal laser-scanning microscopy images of intracellular co-localization between PPT1 and lysosomes. LAMP-1 staining indicates the lysosomes, and Hoechst 33,258 indicates the nucleus. Co-localization is visualized by color and area overlap (red + green = yellow). Scale bar, 10 μm. **B** Fluorescence co-localization analysis according to (**A**) was performed by ImageJ software. **C** Immunofluorescence staining of PPT1 in HCC cells after treatment with DC661 (3 µM, 6 h). Scale bar, 20 μm. **D** Semiquantitative analysis of the mean fluorescence intensity (MFI) in the HCC cells according to (**C**) was performed by using ImageJ software (n = 6). Data represent mean ± SD; **P < 0.01. **E** Fluorescence images of LysoTracker Green (lysosome probe) in HCC cells after treatment with DC661 (3 µM, 6 h). Hoechst 33,258 was used to stain the nucleus. Scale bar, 50 μm. **F** Semiquantitative analysis of the MFI of LysoTracker Green in HCC cells according to (**E**) was performed by ImageJ software (n = 6). Data represent mean ± SD; **P < 0.01. **G** Western blot showing an increase in LC3-II and P62 in HCC cells treated with DC661 (3 µM, 6 h). **H** Western blot showing P62 degradation and LC3 lipidation in HCC cells treated with sorafenib and/or DC661. HCC cells were treated with or without 10 µM sorafenib in the presence or absence of 1 µM DC661 for 24 h

The autophagy process can basically be divided into an early and late stage [9]. The early autophagy stage includes the initiation, nucleation, and elongation of autophagosomes [10], while lysosomes fuse with autophagosomes, hydrolyze damaged organelles, and recycle them during the late stage of autophagy [10, 19]. Because both normative and non-normative autophagies depend on lysosomal degradation, lysosomes provide the best targets for autophagy inhibition [9]. To verify whether PPT1 is associated with lysosomes, we performed GSEA. Interestingly, GSEA confirmed that PPT1 expression was associated with the “KEGG_LYSOSOME” gene set. To explore whether PPT1 is located in lysosomes, we performed immunofluorescence double-labeling of PPT1 and lysosomal-associated membrane protein 1 (LAMP1). The confocal laser microscopy images and further immunofluorescent co-localization analysis confirmed that PPT1 co-localized with LAMP1 (Fig. 3A, B). These results indicated that PPT1 was located in the intracellular lysosomes. When Hep 3B and Hep 1**–**6 were treated with the PPT1 inhibitor DC661, the expression of PPT1 in the DC661-treated group was at significantly lower levels than that in the control group (Fig. 3C, D). To further test whether DC661 inhibited lysosomes, the LysoTracker Green probe was used. Compared with the control group, DC661 treatment resulted in a significant increase in lysosomal deacidification (Fig. 3E, F), which correspondingly inhibited lysosomal degradation of autophagosomes.

Interestingly, we found that treatment with DC661 reduced the expression levels of mTOR (Additional file 1: Fig. S4). DC661 inhibited mTOR and promoted early autophagy, leading to LC3-I increase and conversion to LC3-II. Furthermore, DC661 simultaneously inhibited lysosome to deacidify the lysosome and reduced autophagosomal degradation, which is equivalent to inhibiting late autophagy, leading to the accumulation of LC3-II. Inhibition of the lysosomes reduced the degradation of LC3-II but did not lead to a change in LC3-I, which is a reasonable explanation for the increased expression of both LC3-I and LC3-II in cells after DC661 treatment that also led to the accumulation of P62 (Fig. 3G). Hep 3B and Hep 1-6 cells also demonstrated a dose-dependent response to DC661 (Fig. 3G). Consistently, through the experiment of Ad-mCherry-GFP-LC3 adenovirus infection, we further confirmed that the PPT1 inhibitor DC661 promoted early autophagy by inhibiting mTOR and inhibited late autophagy by inhibiting lysosomes (Additional file 1: Fig. S5). In addition, when cells were treated with sorafenib and DC661 simultaneously, P62 expression was increased compared with the sorafenib-only treatment group. Our findings showed that DC661 blocked the autophagy induced by sorafenib (Fig. 3H).

### Potential molecular mechanism of lysosomal membrane hyperpermeability induced by PPT1 inhibitor

**Fig. 4:**
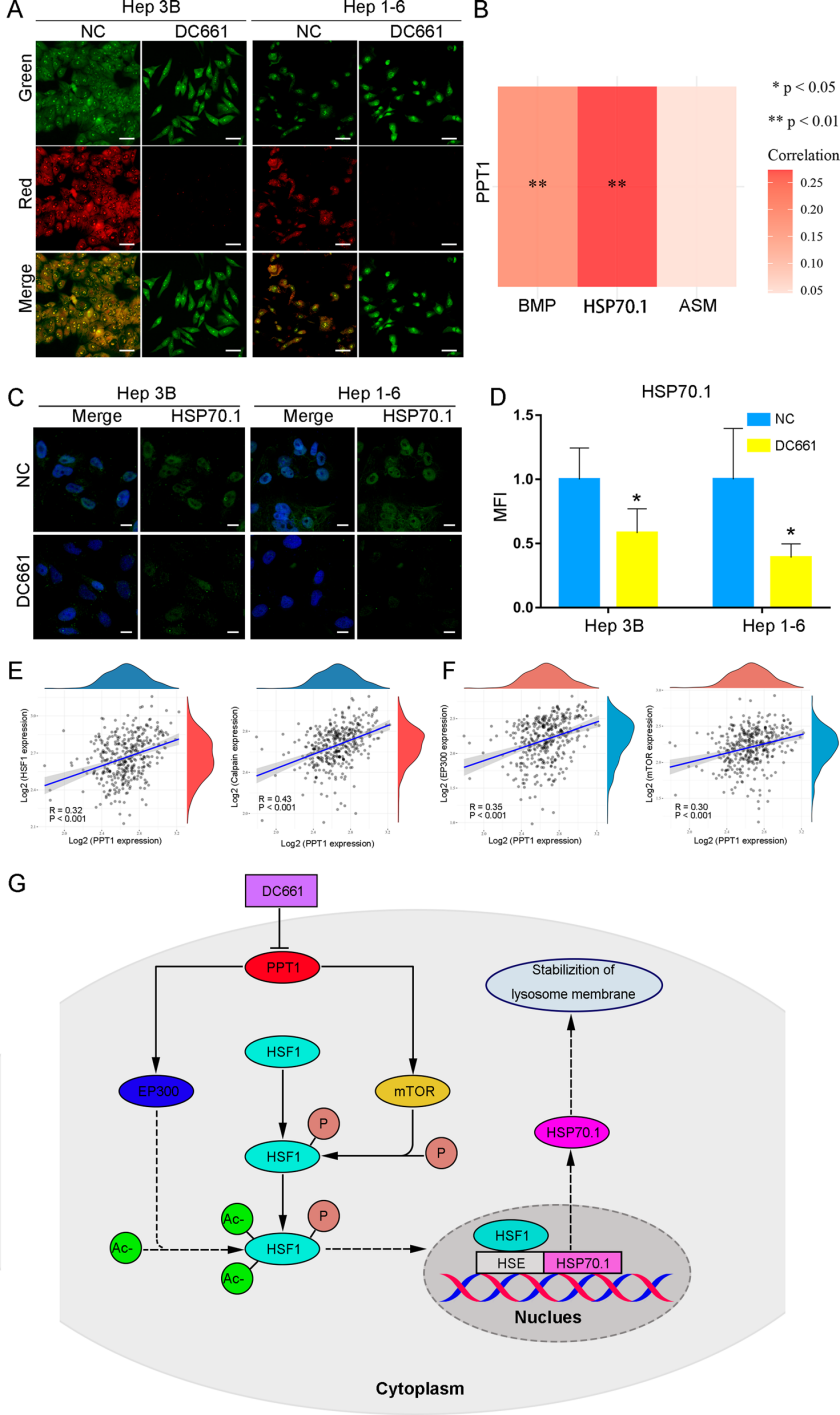
Potential molecular mechanism of lysosomal membrane hyperpermeability induced by PPT1 inhibitor. **A** Fluorescence images of acridine orange staining in HCC cells treated with DC661 (3 µM, 6 h). Hoechst 33,258 staining marks the nuclei. Scale bar, 50 μm. **B** Gene correlation analysis using TCGA dataset. Heat map of the correlation between multiple genes and one gene. PPT1 expression was positively correlated with HSP70.1 and BMP expression in HCC (linear regression); **P < 0.01. **C** Immunofluorescent staining of HSP70.1 in HCC cells treated with DC661 (3 µM, 6 h). Scale bar, 10 μm. **D** Semiquantitative analysis of the mean fluorescence intensity (MFI) in HCC cells according to (**C**) was performed by using ImageJ software (n = 6). Data represent mean ± SD; *P < 0.05. **E** Gene correlation analysis using TCGA dataset. PPT1 expression was positively correlated with HSF1 and calpain expression in HCC (linear regression); *P* < 0.001. **F** Gene correlation analysis using TCGA dataset. PPT1 expression was positively correlated with EP300 and mTOR expression in HCC (linear regression); *P* < 0.001. **G** Schematic illustration of a potential molecular mechanism of lysosomal membrane hyperpermeability induced by PPT1 inhibitor. HSE: heat shock element

To demonstrate whether the PPT1 inhibitor DC661 can induce lysosomal membrane permeability (LMP) and cause lysosomal deacidification, we employed acridine orange (AO) to test lysosomal membrane stability [20]. In the Hep 3B and Hep 1-6 cell lines treated with DC661 and stained with AO, the red spots were significantly reduced compared with the control cells (Fig. 4A), indicating that the PPT1 inhibitor caused lysosomal deacidification by inducing LMP. However, the mechanism by which DC661 influences lysosomal function by enhancing LMP remains unclear, thus we further explored the potential molecular mechanism of lysosomal membrane hyperpermeability induced by PPT1 inhibition.

The HSP70.1/BMP/ASM pathway is considered a major regulatory pathway in lysosomal membrane stability [21, 22]. Binding of HSP70.1 to the lysosomal phospholipid, BMP, has recently been shown to stabilize lysosomal membranes by enhancing the activity of ASM in cancer cells [22]. Sphingomyelin is the main lipid component of plasma membranes and endosomal/lysosomal membranes [21], and ASM plays an important role in membrane lipid turnover by hydrolyzing sphingolipids into phosphocholine and ceramide [21], which stabilizes the lysosomal membrane. To explore the relationship between PPT1 and the HSP70.1/BMP/ASM pathway, gene correlation analysis was performed, which showed that PPT1 expression was positively correlated with HSP70.1 and BMP, but not with ASM (Fig. 4B). As mentioned earlier, the combination of HSP70.1 and BMP enhanced the activity of ASM but did not affect its expression [21]. Our findings provided a preliminary indication that PPT1 function might be closely related to the HSP70.1/BMP/ASM pathway.

We further investigated whether PPT1 inhibitor induced LMP by affecting HSP70.1/BMP/ASM pathway in vitro. HSP70.1 is considered to have an important role in the HSP70.1/BMP/ASM pathway [23]. Interestingly, our results confirmed that PPT1 inhibitor DC661 significantly reduced HSP70.1 expression levels in HCC cells (Fig. 4C, D, Additional file 1: Fig. S6). However, the level of HSP70.1 expression reflects the combined effects of synthesis and degradation. Heat shock transcription factor 1 (HSF1), a major transcription factor regulating stress reactions, is activated under stress to promote the transcription of HSP70.1. Previous studies have shown that the carbonylation of HSP70.1 caused by artificial oxidative stress (such as from hydroxynonenal or H_2_O_2_) is more readily degraded by calpain [21]. To explore whether PPT1 is related to HSF1 and calpain, we carried out genetic correlation analysis and found that PPT1 was positively correlated with HSF1 and calpain (Fig. 4E), indicating that PPT1 inhibitor might inhibit both HSP70.1 synthesis and degradation. Therefore, it is reasonable to suggest that PPT1 inhibitor reduces the expression of HSP70.1 by regulating HSF1. Consistently, our results confirm that DC661 reduced HSF1 expression and further decreased mRNA expression levels of HSP70.1 in HCC cell lines (Additional file 1: Figs. S7, S8).

Next, we further explored the mechanism of how DC661 regulates HSF1 expression. Studies have shown that post-translational modifications (such as acetylation or phosphorylation) serve important functions in regulating HSF1 activity [24]. In the absence of stress, HSF1 homeostasis levels are regulated by histone acetyltransferase EP300, which acetylates specific lysine residues (Lys208 and Lys298) to prevent proteasome degradation and, thus, promote the stability of HSF1 [25]. Studies have shown that silencing EP300 could lead to a decrease in HSF1 protein levels [25]. Previous studies have shown that mTOR directly phosphorylates HSF1 at serine 326, an important residue for transcriptional activation [26]. Interestingly, we found that PPT1 was positively correlated with EP300 and mTOR by gene correlation analysis (Fig. 4F). As shown in Additional file 1: Fig. S4, we confirmed that DC661 decreased the expression levels of mTOR in HCC cells. In addition, PPT1 inhibitor DC661 prevented HSF1-Ser326 phosphorylation (Additional file 1: Fig. S9), and might therefore reduce the transcriptional activity of HSF1. Therefore, we further investigated whether PPT1 inhibitor DC661 could affect the expression level of EP300 in vitro. Consistently, our results confirm that DC661 reduced EP300 expression (Additional file 1 Fig. S10), and might therefore decrease the expression level of HSF1. Based on the above findings, we could reasonably hypothesize that PPT1 inhibitor DC661 reduces HSP70.1 expression levels by downregulating EP300 and mTOR and reducing the expression level and transcriptional activity of HSF1 (Fig. 4G). Moreover, to further confirmed the mechanistic involvement of PPT1, a PPT1-knockdown Hep 3B cell model was constructed by transfection of siRAN (Additional file 1: Fig. S11) and compared with signaling changes in siNC and siPPT1 cells. Our results confirmed that the effects of PPT1 knockdown on lysosomal membrane permeability and signal pathway were consistent with PPT1 inhibitor DC661 (Additional file 1: Fig. S12). However, the exact molecular mechanism by which PPT1 regulates lysosomal membrane stability remains to be further elucidated.

### Mechanism of cell apoptosis induction by DC661

There is growing interest in elucidating the underlying mechanisms that induce cell death, particularly apoptosis. The mitochondria-mediated apoptosis is considered a major pathway in tumor apoptosis. Considering that DC661 is lysosomal-targeting, we investigated the role of mitochondrial apoptosis pathways in the DC661-mediated LMP-induced apoptosis of HCC cells.

**Fig. 5:**
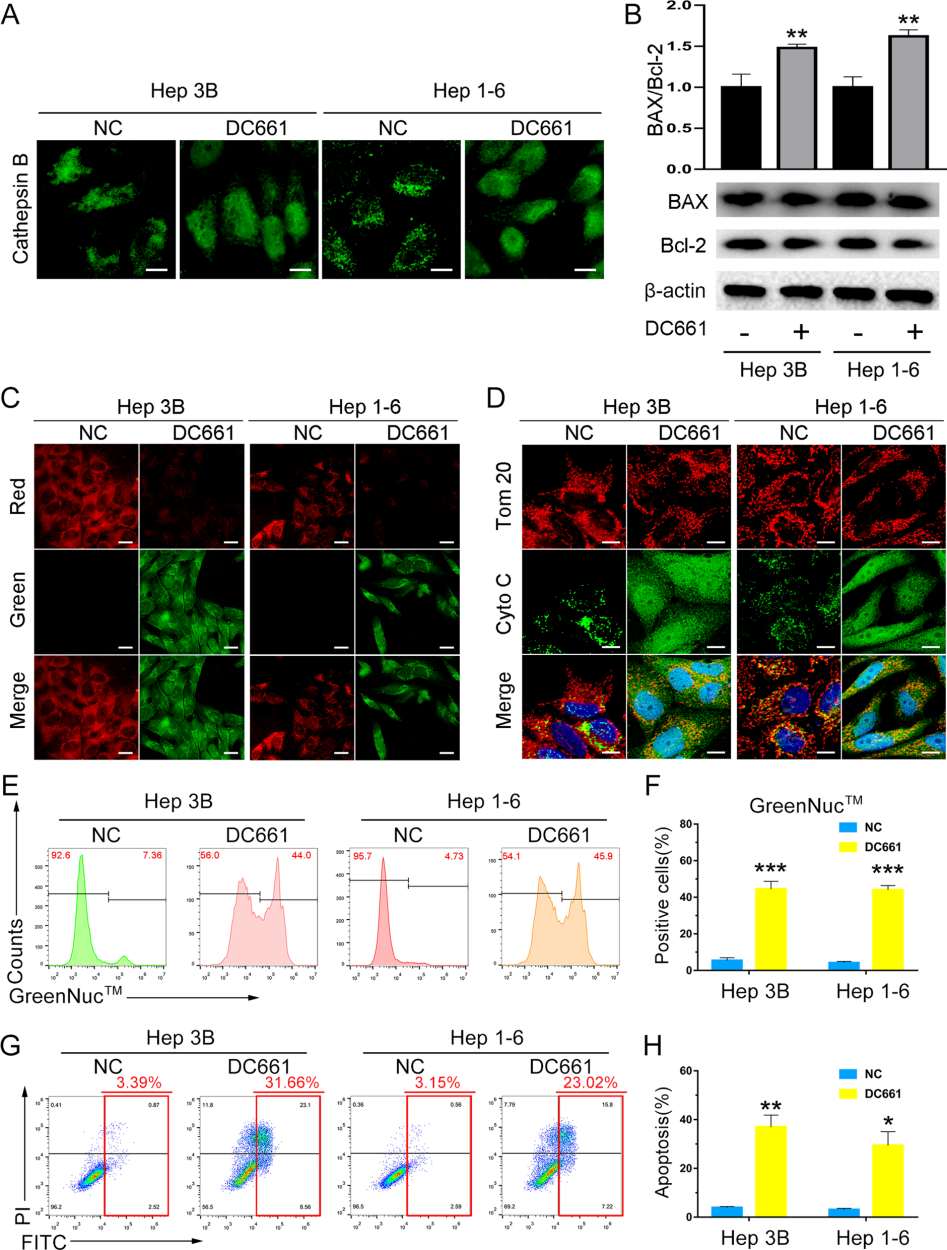
The mechanism of DC661-induced cell apoptosis. **A** Immunofluorescent staining of cathepsin B in HCC cells treated with DC661 (3 µM, 6 h). Representative cells showing that DC661 treatment led to leakage of lysosomal cathepsin B are shown. Scale bar, 10 μm. **B** Western blot showing the level of Bax and Bcl-2 in HCC cells treated with DC661 (3 µM, 6 h). Data represent mean ± SD; **P < 0.01. **C** Effects of DC661 treatment on mitochondrial membrane potential (ΔΨ_m_). Cells were incubated with DC661 at 3 µM for 6 h, then stained with JC-1. Hoechst 33,258 staining indicates the nuclei. Scale bar, 20 μm. **D** Immunofluorescence co-staining of cytochrome c and Tom20 (indicates the mitochondria). Cells were incubated with DC661 at 3 µM for 6 h. Hoechst 33,258 staining indicates the nuclei. Scale bar, 20 μm. **E**, **F** Flow cytometric analysis and statistical data of the level of activated caspase-3 in HCC cells treated with DC661 (1 µM, 24 h). Data represent mean ± SD; ***P < 0.001. **G**, **H** Flow cytometric analysis and statistical data of cell apoptosis and necrosis in HCC cells treated with DC661 (1 µM, 24 h). Data represent mean ± SD; *P < 0.05; **P < 0.01

The leakage of lysosomal cathepsin caused by LMP is the first event that induces apoptosis. As shown in Fig. 5A, DC661 therapy could induce LMP, which destroyed the lysosomal integrity of HCC cells and led to leakage of lysosomal cathepsin B. LMP and the release of cathepsin are associated with the activation of Bax, a proapoptotic member of the Bcl-2 family that is generally associated with regulators of cell death via endogenous apoptotic pathways [20]. Bcl-2, an antiapoptotic member, and Bax are the most significant regulators of apoptosis, for which the Bax to Bcl-2 expression ratio is critical [27]. As shown in Fig. 5B, the Bax to Bcl-2 expression ratio significantly increased after DC661 treatment, indicating the promotion of cell apoptosis. The loss of mitochondrial membrane integrity and the release of cytochrome c are secondary events characterizing the mitochondrial apoptosis pathway. We used the commercial fluorescent probe JC-1 to monitor the mitochondrial membrane potential (ΔΨ_m_), which decreased significantly after DC661 treatment compared with the control group (Fig. 5C). The immunofluorescent analysis of cytochrome c and Tom20 showed that DC661 therapy triggered the release of cytochrome c from the mitochondria (Fig. 5D) and was consistent with the Western blot results (Additional file 1: Fig. S13). Cytochrome c triggered the activation of caspase-3, which is the third event leading to apoptosis through mitochondria-mediated pathways. The results of Western blot showed that DC661 therapy significantly increased the level of cleaved caspase-3 compared with the control group (Additional file 1: Fig. S14). We further explored whether caspase-3 was activated in response to DC661. As shown in Fig. 5E and F, compared with the control group, caspase-3 activity was significantly increased in Hep 3B and Hep 1-6 cells treated with DC661.

All of these results confirmed our hypothesis that DC661-targeting of PPT1 induced apoptosis through a mitochondria-mediated pathway. In brief, DC661 induces LMP and leads to lysosomal rupture, and lysosomal cathepsin disperses throughout the cytoplasm. Bax is activated and the membrane potentials of the mitochondria are depolarized. Finally, cytochrome c is released and caspase-3 is activated, leading to cell apoptosis (Fig. 5G, H).

### Inhibition of PPT1 enhanced anti-tumor immune response

**Fig. 6:**
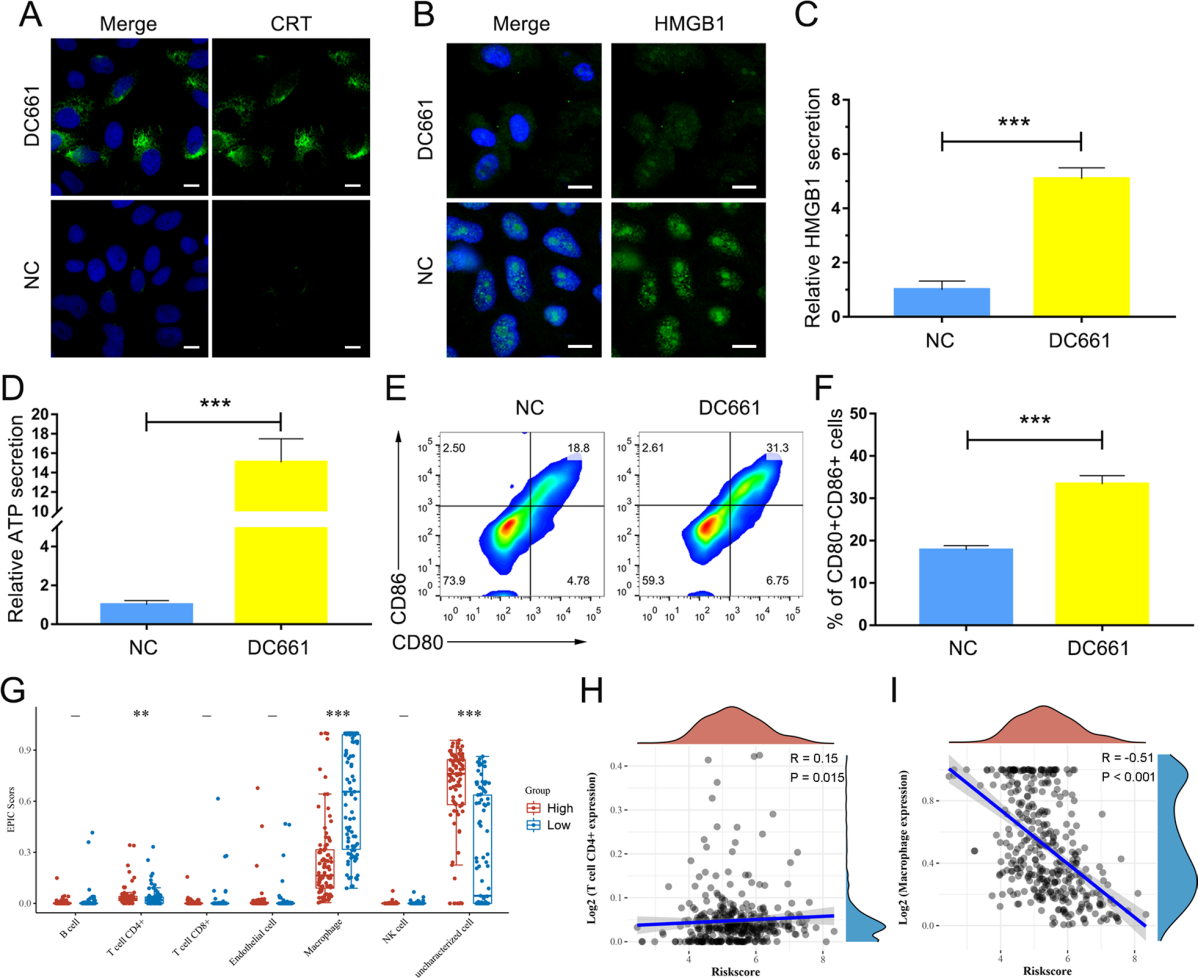
PPT1 inhibitor DC661-induced immunogenic cell death promoted the maturation of DCs and the activation of CD8^+^ T cells. **A** Immunofluorescent imaging of CRT expression on the cell surface of Hep 1-6 cells treated with DC661 (3 µM, 6 h). Nuclei are stained with Hoechst 33,258. Scale bars, 10 μm. **B** Immunofluorescent imaging of HMGB-1 release by Hep 1-6 cells treated with DC661 (3 µM, 6 h). Nuclei are stained with Hoechst 33,258. Scale bars, 10 μm. **C** ELISA detection of HMGB1 release into cell culture medium. Cells were incubated with DC661 at 3 µM for 6 h. Data represent mean ± SD; ***P < 0.001. **D** ELISA detection of ATP release into the cell culture medium. Cells were incubated with DC661 at 3 µM for 6 h. Data represent mean ± SD; ***P < 0.001. **E**, **F** Flow cytometric analysis (left) and quantification (right) of mature DC cells (CD11c^+^CD80^+^CD86^+^) from the spleens of DC661- or vehicle-treated Hep 1-6 tumor-bearing mice. Data represent mean ± SD; ***P < 0.001. **G–I** The expression level of PPT1 was associated with the immune infiltration in the HCC tumor microenvironment. Scatter plots (**G**) and correlation diagrams (**H**, **I**) showing the difference of CD4^+^ T cells and macrophages infiltration level between PPT1-high and -low groups in TCGA-LIHC. **P < 0.01; ***P < 0.001

To further explore whether PPT1 inhibitor could enhance anti-tumor immune response, we first investigated whether DC661 induced immunogenic cell death (ICD) [28]. Previous studies have shown that, at the molecular level, the immunological silhouette of these cells death pathways is defined by a set of molecules called damage-associated molecular patterns, including calreticulin (CRT), high-mobility group box-1 (HMGB1) protein, and adenosine triphosphate (ATP) [29]. CRT is a unique biomarker exposed on the surface of cells undergoing ICD [28, 30, 31]. Furthermore, under normal conditions, CRT is mainly located in the endoplasmic reticulum and is transported to the cell surface in the event of endoplasmic reticulum stress, where it serves as an indicator of ICD [28, 31]. Once exposed on the cell surface, CRT acts as an “eat me” signal, stimulating immature dendritic cells (DCs) and macrophages to engulf dying tumor cells and their apoptotic fragments [28, 31]. When secreted into the intercellular stroma, HMGB1 and ATP act as “find me” signals that are rapidly recognized by phagocytic cells [29]. First, we evaluated the CRT expression of Hep 1-6 cells treated with DC661, which showed a significant amount of surface CRT in the immunostaining and flow cytometric analyses (Fig. 6A, Additional file 1: S15). In addition, we investigated whether DC661 could induce the release of HMGB1 and the secretion of ATP. As shown in Fig. 6B, HMGB1 was transferred from the nucleus to the cytoplasm, and the amount was reduced by DC661. Consistent with the ELISA results, DC661 induced an almost 5-fold extracellular release of HMGB1 compared to the control group (Fig. 6C). In addition, DC661 significantly enhanced ATP secretion by Hep 1-6 cells (Fig. 6D). The CRT exposure and HMGB1 and ATP efflux induced by DC661 were consistently associated with activation of the ICD pathway [32].

We further examined the ICD-induced anti-tumor immune response by measuring DC maturation in vivo [32]. DCs are specialized antigen-presenting cells that present antigens to CD8^+^ cytotoxic T lymphocytes and further activate CD8^+^ cytotoxic T cells [33]. Therefore, the maturation of DCs after DC661 treatment was most likely because of stimulation from tumor-associated antigens released by the PPT1 inhibitor induced ICD. Interestingly, we observed the significant maturation of DCs, as indicated by enhanced CD11c^+^CD80^+^CD86^+^ cells in the spleens of tumor-bearing mice treated with DC661 (Fig. 6E, F). To further validate the maturation of DCs after DC661 treatment, we investigated the serum level of IL-12 produced by mature DCs upon antigen stimulation. There was a significant upregulation of IL-12 levels in the DC661 group compared with the control groups (Additional file 1: Fig. S16), confirming that DC maturation was triggered by DC661 treatment.

We found administration of DC661 for 7 days in vivo clearly increased the number of activated CD8^+^ T lymphocytes and activated CD4^+^ T lymphocytes in tumors (Additional file 1: Figs. S17, S18). Furthermore, when we detected the levels of IFN-γ secreted by activated T lymphocytes into tumor tissues, there was a significant upregulation of IFN-γ levels in the DC661 group compared with the control groups (Additional file 1: Fig. S19). Interestingly, DC661 reduced the infiltration of tumor myeloid-derived suppressor cells (MDSCs, CD45^+^CD11b^+^Gr-1^+^), which play a major immunosuppressive role in the tumor microenvironment (Additional file 1: Fig. S20). In addition, the correlation between the expression level of PPT1 and immune cell infiltration level was analyzed by spearman correlation in TCGA-LIHC [34]. Interestingly, we found that the expression of PPT1 was positively correlated with the abundance of acquired immunocytes (CD4^+^ T lymphocytes), and negatively correlated with the abundance of innate immunocytes (tumor-associated macrophages) (Fig. 6G**–**I). According to the above research results, we confirmed that the expression level of PPT1 was associated with the immune infiltration in the HCC tumor microenvironment and PPT1 inhibitor DC661 could enhance anti-tumor immune response.

### DC661 overrode adaptive resistance to sorafenib in vitro

**Fig. 7:**
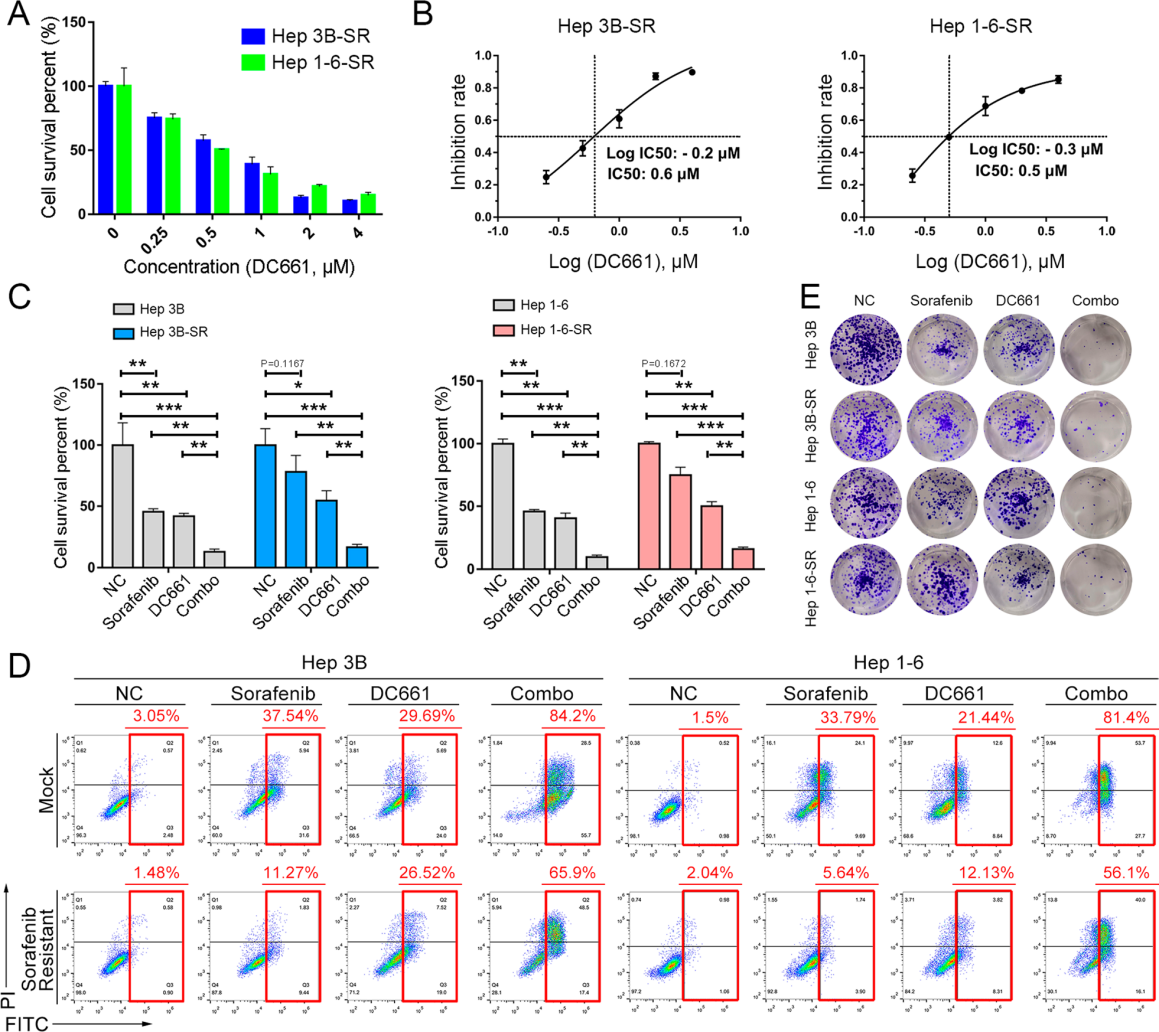
DC661 overrode adaptive resistance to sorafenib in vitro. **A** Cell viability after treatment with different DC661 concentrations for 48 h was determined by CCK-8 assay. **B** IC50 values for DC661 in Hep 3B-SR and Hep 1-6-SR cells according to (**A**) were determined by CCK-8 assay. The data shown are from three independent experiments. **C** Cell viability in HCC cells after treatment with sorafenib (1.5 µM) and/or DC661 (0.5 µM) for 48 h was determined by CCK-8 assay. *P < 0.05; **P < 0.01; ***P < 0.001. **D** Flow cytometric analysis of cell apoptosis and necrosis in HCC cells treated with sorafenib (1.5 µM) and/or DC661 (0.5 µM) for 48 h. **E** For the colony formation assay after sorafenib (1.5 µM, 48 h) and/or DC661 (0.5 µM, 48 h) therapy, HCC cells were first seeded into a 6-well plate at a density of 1000 cells/well and routinely cultured for 14 days, then stained with crystal violet and imaged

To explore the effect of DC661 on HCC cells, we selected Hep 3B and Hep 1-6 cell lines with high PPT1 expression (Fig. 1D). The CCK-8 test showed DC661 inhibited the growth of the two HCC cell lines in a dose-dependent manner (Fig. 7A), with the highest IC50 values of 0.6 and 0.5 µM, respectively (Fig. 7B). We then examined the combined effects of DC661 and sorafenib on the HCC cells. According to CCK-8, DC661 caused Hep 3B and Hep 1-6 cells to be sensitive to sorafenib treatment (Fig. 7C). In the Annexin-V apoptosis assay, treatment with DC661 and sorafenib synergistically induced apoptosis in two HCC cell lines (Fig. 7D). In addition to the increased percentage of apoptotic cells, the combination-treated HCC cells exhibited inhibited proliferation in a synergistic manner, as shown by the decrease in clone formation (Fig. 7E, Additional file 1: Fig. S21). To explore the sorafenib-resistance reversal effect of DC661 on HCC cells, we treated Hep 3B-SR and Hep 1-6-SR cells with DC661 and sorafenib simultaneously. Hep 3B-SR and Hep 1-6-SR cells consistently showed less inhibition of apoptotic cells and clone formation than the control cells after sorafenib administration (Fig. 7C–E). Even more strikingly, DC661 synergistically reversed the sorafenib-resistance phenotype of Hep 3B-SR and Hep 1-6-SR cells (Fig. 7C–E). Moreover, PPT1 knockdown enhanced sorafenib sensitivity in HCC cells (Additional file 1: Fig. S22).

### DC661 combined with sorafenib resulted in maximal tumor growth suppression in HCC tumor models

**Fig. 8:**
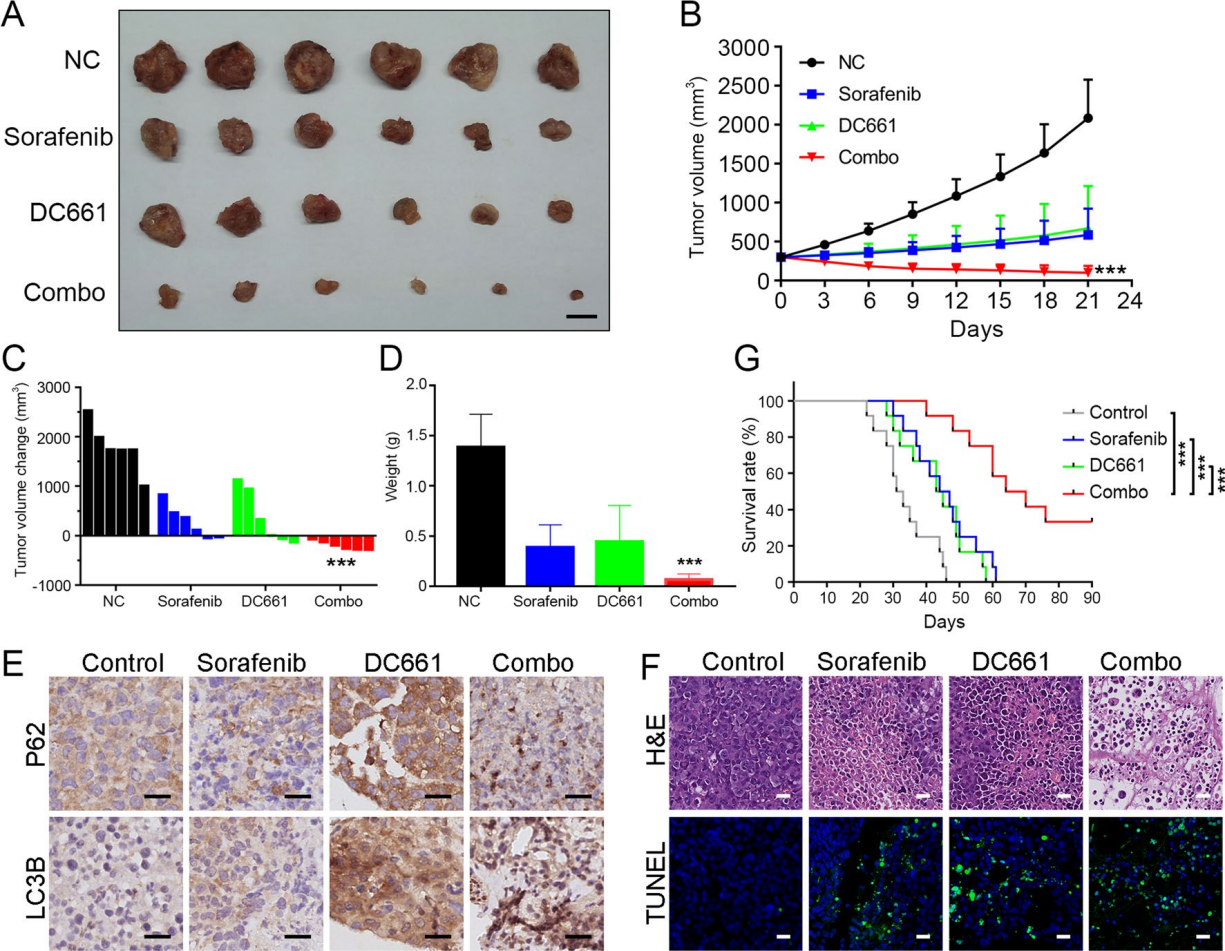
DC661 combined with sorafenib resulted in maximal tumor growth suppression in HCC tumor models. **A** Response of Hep 1-6-SR cell derived HCC tumors to treatment with DC661 (3 mg/kg/d), sorafenib (30 mg/kg/d), or both drugs (DC661 3 mg/kg/d, sorafenib 30 mg/kg/d). The tumor after 21 days of treatment is shown. Scale bar, 1 cm. **B** Graph showing the volume of tumors after 21 days of treatment; ***P < 0.001, Mann-Whitney U test. **C** Waterfall plot showing the response of each tumor after 21 days; ***P < 0.001, Mann-Whitney U test. **D** Graph showing the weight of tumors after 21 days of treatment; ***P < 0.001, Mann-Whitney U test. **E** Immunohistochemical images of LC3B and P62 in resected tumors. Scale bar, 20 μm. **F** H&E staining for pathological changes in tumor sections (top row). TUNEL staining (green) for apoptosis in tumor sections (bottom row). Blue fluorescence indicates the cell nuclei. Scale bar, 20 μm. **G** Survival curves for four groups of tumor-bearing mice given different treatments (n = 12 per group); ***P < 0.001, Log-rank test

We examined the therapeutic effect of DC661 treatment alone and its combined effect with sorafenib in vivo using Hep 1-6-SR cell derived HCC tumors of a uniform tumor size. The tumors and their corresponding volumes are shown in Fig. 8A, B. After treatment for 21 days, DC661 reduced tumor volumes in a manner similar to sorafenib, and DC661 combined with sorafenib exerted a synergistic effect, resulting in maximal suppression of the tumor growth compared with the control group. We found that the combination treatment markedly reduced the tumor volumes of the Hep 1-6-SR cell derived HCC tumors relative to the original tumor volume on day 1 of treatment (Fig. 8C). As anticipated, a significant difference in tumor weight was observed between the combined treatment group and the other groups (Fig. 8D). Consistent with these biological effects, the autophagy flux induced by sorafenib was blocked by DC661 in the combination treatment group (Fig. 8E). To further investigate the anti-tumor effect of the combined therapy, hematoxylin and eosin (H&E) staining and TUNEL were used to analyze the histological changes and apoptosis levels of in vivo tumors. As shown in Fig. 8F, tumor cells in the combined treatment group showed the largest degree of nuclear deletion, indicating that most tumor cells were destroyed by the combined therapy compared with single-agent treatment and mock controls. The TUNEL analysis showed that the apoptosis level in tumors followed the same trend (Fig. 8F). Additionally, as shown in Fig. 8G, the survival time of mice in the combination therapy group was significantly prolonged (P < 0.001). All these results suggested that DC661 could represent a powerful strategy for reversing sorafenib adaptive resistance and increasing the sensitivity of HCC to sorafenib.

## Conclusions

In conclusion, PPT1 inhibition could impede sorafenib-induced autophagy and enhance the sensitivity of sorafenib, possibly by inhibiting the HSP70.1/BMP/ASM pathway and lysosomes. In addition, the expression level of PPT1 was associated with the immune infiltration in the HCC tumor microenvironment and PPT1 inhibitor DC661 significantly enhanced the anti-tumor immune response. In order to reduce the side effects of PPT1 inhibitor DC661, smart organic nanocarriers with self-adaptive responsiveness for improving tumor drug delivery and curative effect might have broad application prospects [35–38]. Overall, targeting PPT1 with DC661 in combination with sorafenib might be a novel and effective therapeutic strategy against HCC.

## Materials and methods

### Data mining the TCGA database

PPT1 mRNA expression levels in several common cancers (including normal tissues and tumor tissues) were reviewed using GEPIA (http://gepia.cancer-pku.cn*)* [39]. The mRNA-seq and clinical data (level 3) of 50 normal liver tissues and 371 primary HCC tissues were extracted from the TCGA database (TCGA-LIHC data set) (https://cancergenome.nih.gov/*).* Among them, 364 patients had both mRNA-seq data and clinical data. The package edgeR of R was used to normalize the raw count data and identify the differentially expressed genes (mRNA) among the HCC samples and normal controls. |log2 (fold change)| > 1 and an adjusted P-value of < 0.05 were set as the optimum cut-off criteria.

### Gene set enrichment analysis and immune cell infiltration analysis

GSEA was performed to evaluate the correlations between PPT1 expression (high vs. low) and cancer-related pathways using the TCGA dataset. C2 (c2.cp.kegg.v6.0.symbols.gmt) from the Molecular Signatures Database (MSigDB) was selected as the reference gene set [40]. By analyzing with 1000 permutations, a normalized enrichment score was obtained, and a gene set was regarded as significantly enriched when a normal *P*-value was < 0.05 [40].

To make reliable immune infiltration estimations, we utilized the immunedeconv, a R package which integrates six state-of-the-art algorithms, including TIMER, xCell, MCP-counter, CIBERSORT, EPIC and quanTIseq. The relative tumor infiltration levels of 6 immune cell types were quantified by EPIC to interrogate expression levels of genes in published signature gene lists [41]. To explore the correlation between PPT1 and the infiltration levels of immune cells and the association of infiltration of immune cells with the different expression groups of PPT1, Wilcoxon rank sum test, and Spearman correlation were adopted. Statistical ranking for PPT1 expression above 75% quartile value was defined as PPT1-high group and below 25% quartile value was defined as PPT1-low group, respectively.

### Gene correlation analysis

The dataset used comprised mRNA sequence data for 371 HCC patients from the TCGA database (https://tcga-data.nci.nih.gov/tcga/*).* The multi-gene correlation map was displayed using the R software package pheatmap, and two-gene correlation maps were generated in the R software package ggstatsplot. We used Spearman’s correlation analysis to describe the correlations between quantitative variables without normal distribution [42].

### Human cancer cell lines culture and Clinical Samples

The human HCC cell lines (Hep G2, Hep 3B, and SMMC-7721) and mouse cell lines (Hep 1-6) and normal human liver cell lines (L-O2 and MIHA) were purchased from the Cell Bank of the Chinese Academy of Sciences (Shanghai, China). The cells were cultured in Roswell Park Memorial Institute-1640 (RPMI-1640, Gibco, NY, USA) containing 10% Fetal Bovine Serum (FBS, Gibco, NY, USA) and 1% penicillin–streptomycin at 37 °C in a humidified environment containing 5% CO_2_.

A total of 15 pairs of normal liver tissues (3 cm away from tumor) and HCC tumor tissues were collected from patients and embedded in paraffin to make specimen slices to detect PPT1 protein expression in Union Hospital, Tongji Medical College, HUST, Wuhan, China.

### Establishment of sorafenib-resistant HCC cells

Sorafenib-resistant clones of Hep 3B and Hep 1-6 cell lines were established as previously described [43]. In brief, HCC cells were induced to become resistant to sorafenib (TargetMol, Boston, USA) by dose escalation at low concentration combined with intermittent shock at high dosage. The same volume of dimethyl sulfoxide (DMSO) was added to mock control cells during the establishment of drug-resistant cells. Sorafenib-resistant tumors were established by injecting subcutaneously 3 × 10^6^ sorafenib-resistant Hep 1-6 cells into the right front flanks of C57BL/6 mice.

### Immunoblot analysis

Immunoblot analyses were performed as previously described [35]. For immunodetection, the following primary antibodies were used: anti-PPT1 monoclonal antibody (Ab) (BOSTER, Catalog number: M02690); anti-P62 monoclonal Ab, anti-LC3B monoclonal Ab, and anti-Bcl-2 monoclonal Ab (ABclonal, Boston, USA); anti-HSP70.1 monoclonal Ab, anti-Cathepsin B polyclonal Ab, and anti-cytochrome c monoclonal Ab (Proteintech, Wuhan, China); anti-BAX monoclonal Ab, anti-HSF1 polyclonal Ab, and anti-phospho-HSF1 (Ser307) polyclonal Ab (Affinity Biosciences, Jiangsu, China); anti-caspase 3 monoclonal Ab (Bioswamp, Wuhan, China).

### Transfection with adenovirus expressing mCherry-GFP-LC3B fusion protein

Hep 3B and Hep 1-6 cells were transfected with an adenovirus expressing an mCherry-GFP-LC3B (Ad-mCherry-GFP-LC3B, Beyotime, Shanghai, China) fusion protein according to the manufacturer’s instructions [44]. At 24 h after transfection, the medium was changed to complete medium containing 10 µM sorafenib or 1 µM DC661 (TargetMol, Boston, USA) or 0.1% DMSO, and the cells were further cultured for 24 h and examined under a fluorescence microscope.

### Transmission electron microscopy

After different treatments, Hep 3B and Hep 1-6 cells were fixed with 2.5% glutaraldehyde for 4 h. The cells were carefully washed with 0.1 M PBS three times and post-fixed with 1% osmium tetroxide in 0.1 M PBS for 2 h. After dehydration with a graded alcohol series, the cells were embedded in epoxy resin. Ultrathin sections were stained with uranyl acetate and lead citrate, each for 15 min. Images were captured with an HT7700-SS transmission electron microscope (Hitachi, Tokyo, Japan).

### Quantitative real-time PCR

Quantitative real-time PCR (q-PCR) was performed as previously described [35] using the following primer sequences: HSP70.1 forward primer: 5′-TGGTGCAGTCCGACATGAAG-3′ and reverse primer: 5′-GCTGAGAGTCGTTGAAGTAGGC-3′; PPT1 forward primer: 5′- AGCCGAATACTGGCATGACC-3′ and reverse primer: 5′- TTGATACCCCGCTCCTGATTT-3′; GAPDH forward primer: 5′-GGAGCGAGATCCCTCCAAAAT-3′ and reverse primer: 5′-GGCTGTTGTCATACTTCTCATGG -3′.

### siRNA transfection

As shown in Additional file 1: Table S1, three specific siRNAs targeting PPT1 (siPPT1) and a negative control siRNA duplex (siNC), were designed and synthesized by Shanghai GenePharma Co.,Ltd (China). Subsequently, siPPT1#3, as the most effective option for downregulation of PPT1 expression, was selected for further study (Additional file 1: Fig. S23). Hep 3B cells were transfected with 100 nM siPPT1 or siNC for 48 h in the presence of lipofectamine 3000 (Invitrogen, Carlsbad, USA) according to the manufacturer’s instructions (GenePharma, Shanghai, China).

### Cell growth assay

The CCK-8 assay (Beyotime, Shanghai, China) was used to measure cell viability after different treatments, as previously described [35]. Briefly, cells were first seeded into a 96-well plate at a density of 5000 cells/well. After the different treatments, cells were subjected to 10 µL of CCK8 and incubated in the dark for 3 h at 37 °C. Then, the optical density values at 450 nm were measured on a microplate spectrophotometer (Thermo Scientific, USA). For the colony formation assay after different treatments, cells were first seeded into a 6-well plate at a density of 1000 cells/well and routinely cultured for 14 days. The cells were subsequently fixed with 4% paraformaldehyde for 15 min and then stained with 0.1% crystal violet for 10 min. The colony formation images were captured on the camera.

### In vitro caspase-3 assay

The caspase-3 activity of cells was quantified by flow cytometry analysis using the caspase-3 activity detection kit for live cells (Beyotime, Shanghai, China) in accordance with the manufacturer’s protocol [45]. Briefly, after the different treatments, the cell culture medium was transferred to a suitable centrifuge tube, and the cells in the 6-well plate were collected using trypsin-EDTA solution (Boster Biological Technology Co. Ltd., CA, USA) and transferred to the same centrifuge tube. The cells were separated from the suspension by centrifugation at 1000 rpm for 5 min. After being carefully washed twice with PBS, the cells were incubated with 5 µM of GreenNuc Caspase-3 substrate in the dark for 30 min at 25 °C. The cell samples were immediately analyzed on a flow cytometer (BD, CA, USA).

### Cell apoptosis

The proportion of apoptotic cells was quantified by flow cytometry analysis using the Annexin V-FITC/PI Apoptosis Assay Kit (Boster, CA, USA). Flow cytometry analysis was performed as previously described [35].

### LysoTracker Green staining

After different treatments, cells were washed with fresh DMEM and incubated with 75 nM LysoTracker Green for 1 h at 37 °C (Yeasen Biotech, Shanghai, China). Nuclei were stained with Hoechst 33,258 (Servicebio, Wuhan, China), and images were captured with a fluorescence microscope.

### Lysosomal membrane stability

Lysosomal membrane stability was tested using AO (Sigma-Aldrich, Shanghai, China). After different treatments, cells were incubated with AO solution (5 µg/mL) in complete medium for 15 min at 37 °C, and images were captured with a fluorescence microscope.

### Mitochondrial membrane potential measurement

The mitochondrial membrane potential (ΔΨ_m_) of cells was detected with JC-1 from the mitochondrial membrane potential assay kit (Beyotime, Shanghai, China) in accordance with the manufacturer’s protocol. Briefly, after different treatments, cells in the 12-well plate were incubated with 500 µL of complete medium and 500 µL of JC-1 dyeing working fluid for 20 min at 37 °C. After washing twice with precooled JC-1 staining buffer, the cells were observed and photographed under fluorescence microscopy.

### Immunofluorescence analysis

Immunofluorescence analyses were conducted as previously described [35]. No cell-permeable fluid was used in the immunofluorescence procedures for proteins located on the surface of cell membranes. Images were captured with a fluorescence microscope, and relative quantitative fluorescence analysis was performed using ImageJ software.

### Immunofluorescence co-staining analysis

Immunofluorescence co-staining was performed as previously described [35]. Briefly, the cells were incubated with mouse monoclonal PPT1 Ab (1:50) and rabbit polyclonal LAMP1 Ab (1:50) overnight at 4 °C. Then, the cells were incubated with phycoerythrin PE-conjugated anti-mouse secondary antibody and FITC-conjugated rabbit secondary antibody (1:100, Boster Biological Technology Co., Ltd.) in the dark for 1 h. Nuclei were stained with Hoechst 33,258 (Servicebio, Wuhan, China), and images were captured with confocal laser scanning microscopy. Immunofluorescence co-staining for cytochrome c and Tom20 was performed using the same method. Fluorescence co-localization analysis was performed using ImageJ software [46].

### ELISA analysis

The respective media and supernatant of cells and tumor tissues after different treatments were collected for ELISA analysis of HMGB1, APT, IL-12, and IFN-γ using the appropriate ELISA kits and following the manufactures’ instructions.

### In vivo DC maturation assays

To study the effects of DC661 treatment on DC maturation in vivo, a Hep 1-6 subcutaneous tumor model grown in C57BL/6 mice was used. When the tumors grew to about 300 mm^3^, the mice were treated by intraperitoneal injection of PBS (control) or DC661. The spleens of tumor-bearing mice were collected 7 d after the different treatments, and DCs were analyzed by flow cytometry. Mature DCs were defined as CD11c^+^CD80^+^CD86^+^ cells [30].

### In vivo tumor immune microenvironment assays

To study the effects of DC661 treatment on the tumor immune microenvironment *in vivo*, a Hep 1-6 subcutaneous tumor model in C57BL/6 mice was used. When the tumors grew to about 300 mm^3^, the mice were treated by intraperitoneal injection of PBS or DC661 (3 mg/kg/d; TargetMol). Tumor tissues were collected 7 d after treatment and digested to form a single-cell suspension using a tumor digestion kit according to the manufacturer’s protocol. After lysing the red blood cells, a single-cell suspension of tumor tissue was analyzed by flow cytometry. Activated CD8^+^ T cells were defined as CD3^+^CD8^+^CD69^+^ cells, and activated CD4^+^ T cells were defined as CD3^+^CD4^+^CD69^+^ cells [47]. MDSCs were defined as CD45^+^CD11b^+^Gr-1^+^ cells [30].

### In vivo drug treatment assay

All animal experiments were performed in compliance with the guidelines of the Animal Care Committee at Tongji Medical College, Huazhong University of Science and Technology (HUST, IORG number: IORG0003571), and the study was performed in accordance with the declaration of Helsinki. In total, 5 × 10^6^ sorafenib-resistant Hep 1-6 cells were injected subcutaneously into the right front flanks of C57BL/6 mice. Once the tumors were established and reached approximately 300 mm^3^, the mice were randomly divided into four groups (n = 18, in each group) treated with DMSO, sorafenib (30 mg/kg/d; TargetMol), DC661 (3 mg/kg/d; TargetMol), or sorafenib and DC661 combined. The mice were given sorafenib orally, while DC661 was administered intraperitoneally, both on a daily basis. Tumor dimensions were measured using a vernier caliper every 3 days and the volume calculated using the following formula: volume (cubic centimeters) = L × W × W × 0.5. The mice (n = 6, in each group) were treated for 21 days before sacrifice, at which point tumors were harvested and the volume and weight calculated. The survival times of the rest of the mice in each treatment group (n = 12) were recorded every other day.

### Hematoxylin and eosin staining

H&E staining was performed as previously described [35], and images were captured using optical microscopy (Olympus, Japan).

### TUNEL assay

The TUNEL assay was performed as previously described [35]. DAPI was used to stain the nuclei, and images were captured by a fluorescence microscope.

### Immunohistochemical analysis

Immunohistochemical staining was performed as previously described [35], and images were captured using optical microscopy (Olympus, Japan). Each tissue slice was assigned a score based on the proportion of stained cells (0 = 0%, 1 = 1–25%, 2 = 25**–**50%, 3 = 50–75%, 4 = 75–100%) and the intensity of the staining (0 = no staining, 1 = weak staining, 2 = moderate staining, and 3 = strong staining) as previously described [48].

### Statistical analysis

The PPT1 expression levels in HCC tissues were distributed according to quartile. Survival analyses for PFS and OS were performed by utilizing the Kaplan–Meier method and log-rank test. A Cox proportional hazards model was performed to evaluate the relative risk factors associated with OS, with hazard ratios (HR) and 95% confidence intervals (95% CI) obtained for each variable. All quantitative data were analyzed using Student’s t-test or the Mann-Whitney U test wherever appropriate. The results are shown as the mean and standard deviation, and *P* < 0.05 was considered statistically significant (*P < 0.05, **P < 0.01, ***P < 0.001). The statistical significance of the results was determined by GraphPad Prism.

## Supplementary Information

**Additional file 1:** **Figure S1.** TCGA database-based comparison of PPT1 mRNA expression in the HCC tissues (*n* = 50) and its paired-normal tissues. ***P < 0.001. **Figure S2**. Comparison of PPT1 protein expression in patient-derived HCC tissues (*n* = 15) and its paired-normal tissues. ***P < 0.001. **Figure S3.** Kaplan–Meier curves of progression free survival (PFS). High PPT1 expression is correlated with poor PFS in HCC patients. **Figure S4.** Immunofluorescence staining and semiquantitative analysis of mTOR in Hep 3B and Hep 1-6 cells treated with DC661 (3 μM, 6 h). Scale bar, 20 μm. Data represent mean ± SD; *P < 0.05; ***P < 0.001, compared with control group. **Figure S5.** Photographs of fluorescence microscopy of punctate fluorescence of a transfected mCherry-GFP-LC3 construct in Hep 3B and Hep 1-6 cells after treatment with DC66 (3 μM, 6 h), Hoechst 33258 labels the nucleus. Scale bar, 10 μm. **Figure S6.** Western blot showing HSP70.1 decrease in Hep 3B and Hep 1-6 cells treated with DC661 (3 μM, 6 h). Data represent mean ± SD; *P < 0.05; **P < 0.01, compared with control group. **Figure S7.** Western blot showing HSF1 decrease in Hep 3B and Hep 1-6 cells treated with DC661 (3 μM, 6 h). Data represent mean ± SD; **P < 0.01; ***P < 0.001, compared with control group. **Figure S8.** The expression of HSP70.1 mRNA in Hep 3B and Hep 1-6 cells treated with DC661 (3 μM, 6 h). Data represent mean ± SD; *P < 0.05, compared with control group.  **Figure S9.** Western blot showing p-HSF1(ser 326) decrease in Hep 3B and Hep 1-6 cells treated with DC661 (3 μM, 6 h). Data represent mean ± SD; **P < 0.01, compared with control group. **Figure S10.** Immunofluorescence staining and semiquantitative analysis of EP300 in HCC cells after treatment with DC661 (3 μM, 6 h). Scale bar, 20 μm. Data represent mean ± SD; **P < 0.01, compared with control group. **Figure S11.** Western blot analysis of PPT1 in siNC and siPPT1 Hep 3B cells.  **Figure S12.** The effect of PPT1 knockdown on lysosomal membrane permeability and signal pathway. (A) Fluorescence images of AO staining in siNC and siPPT1 Hep 3B cells. Scale bar, 50 μm. (B,C) Immunofluorescent staining and semiquantitative analysis of HSP70.1 in siNC and siPPT1 Hep 3B cells. Scale bar, 10 μm. Data represent mean ± SD; **P < 0.01. (D) Western blot analysis of HSF1 and p-HSF1 in siNC and siPPT1 Hep 3B cells. (E,F) Immunofluorescent staining and semiquantitative analysis of EP300 in siNC and siPPT1 Hep 3B cells. Scale bar, 20 μm. Data represent mean ± SD; **P < 0.01. (G,H) Immunofluorescent staining and semiquantitative analysis of mTOR in siNC and siPPT1 Hep 3B cells. Scale bar, 20 μm. Data represent mean ± SD; **P < 0.01. **Figure S13.** Western blot analysis of cytochrome c release in Hep 3B and Hep 1-6 cells treated with DC661 (3 μM, 6 h). Data represent mean ± SD; ***P < 0.001, compared with control group. **Figure S14**. Western blot analysis of caspase-3 activation in Hep 3B and Hep 1-6 cells treated with DC661 (3 μM, 6 h).  **Figure S15.** Flow cytometric analyses of CRT expression on cell membrane in Hep 3B and Hep 1-6 cells treated with DC661 (3 μM, 6 h). Data represent mean ± SD; ***P < 0.001, compared with control group. **Figure S16.** IL-12 levels in the serum from tumor-bearing mice after different treatments as measured by Elisa kit. Data represent mean ± SD; ***P < 0.001, compared with control group. **Figure S17.** Flow cytometric analyses of activated CD8+ T cells (CD8+CD69+) from the tumor microenvironment of DC661- or vehicle-treated Hep 1-6 tumor-bearing mice. Data represent mean ± SD; ***P < 0.001, compared with control group.  **Figure S18.** Flow cytometric analyses of activated CD4+ T cells (CD4+CD69+) from the tumor microenvironment of DC661- or vehicle-treated Hep 1-6 tumor-bearing mice. Data represent mean ± SD; **P < 0.01, compared with control group. **Figure S19.** Expression of IFN-γ in tumor microenvironment was detected by Elisa. Data represent mean ± SD; **P < 0.01, compared with control group. **Figure S20.** Flow cytometric analyses of MDSCs (CD45+CD11b+Gr-1+) in tumor. Data represent mean ± SD; **P < 0.01, compared with control group. Myeloid-derived suppressor cells: MDSCs. **Figure S21.** Quantification for the colony formation assay after different treatments. **Figure S22.** Cell viability in untreated or siNC and siPPT1 HCC cells after treatment with sorafenib (1.55 μM, 48 h) was determined by CCK-8 assay. *P < 0.05; **P < 0.01; ***P < 0.001. **Figure S23.** The expression of PPT1 mRNA after transfected with siPPT1. Data represent mean ± SD; **P < 0.01, ***P < 0.001, compared with siNC group. **Table S1.** The sequences for siPPT1.

## Data Availability

The TCGA data referenced in the study are available in a public repository from TCGA website (http://cancergenome.nih.gov/). All data generated and/or analyzed during this study are available from the corresponding author upon reasonable request.
