# Supplementary material for: High PPT1 expression predicts poor clinical outcome and PPT1 inhibitor DC661 enhances sorafenib sensitivity in hepatocellular carcinoma
Source: Cancer Cell Int. 2022 Mar 11;22:115. doi: 10.1186/s12935-022-02508-y (PMC8917761; doi:10.1186/s12935-022-02508-y)
Supplement: Supplementary file 1 — Additional file 1: Figure S1. TCGA database-based comparison of PPT1 mRNA expression in the HCC tissues (n = 50) and its paired-normal tissues. ***P < 0.001. Figure S2. Comparison of PPT1 protein expression in patient-derived HCC tissues (n = 15) and its paired-normal tissues. ***P < 0.001. Figure S3. Kaplan–Meier curves of progression free survival (PFS). High PPT1 expression is correlated with poor PFS in HCC patients. Figure S4. Immunofluorescence staining and semiquantitative analysis of mTOR in Hep 3B and Hep 1-6 cells treated with DC661 (3 μM, 6 h). Scale bar, 20 μm. Data represent mean ± SD; *P < 0.05; ***P < 0.001, compared with control group. Figure S5. Photographs of fluorescence microscopy of punctate fluorescence of a transfected mCherry-GFP-LC3 construct in Hep 3B and Hep 1-6 cells after treatment with DC66 (3 μM, 6 h), Hoechst 33258 labels the nucleus. Scale bar, 10 μm. Figure S6. Western blot showing HSP70.1 decrease in Hep 3B and Hep 1-6 cells treated with DC661 (3 μM, 6 h). Data represent mean ± SD; *P < 0.05; **P < 0.01, compared with control group. Figure S7. Western blot showing HSF1 decrease in Hep 3B and Hep 1-6 cells treated with DC661 (3 μM, 6 h). Data represent mean ± SD; **P < 0.01; ***P < 0.001, compared with control group. Figure S8. The expression of HSP70.1 mRNA in Hep 3B and Hep 1-6 cells treated with DC661 (3 μM, 6 h). Data represent mean ± SD; *P < 0.05, compared with control group. Figure S9. Western blot showing p-HSF1(ser 326) decrease in Hep 3B and Hep 1-6 cells treated with DC661 (3 μM, 6 h). Data represent mean ± SD; **P < 0.01, compared with control group. Figure S10. Immunofluorescence staining and semiquantitative analysis of EP300 in HCC cells after treatment with DC661 (3 μM, 6 h). Scale bar, 20 μm. Data represent mean ± SD; **P < 0.01, compared with control group. Figure S11. Western blot analysis of PPT1 in siNC and siPPT1 Hep 3B cells. Figure S12. The effect of PPT1 knockdown on lysosomal membrane permeability and signa [file 12935_2022_2508_MOESM1_ESM.docx]

**Additional file 1**

**High PPT1 expression predicts poor clinical outcome and PPT1 inhibitor DC661 enhances sorafenib sensitivity in hepatocellular carcinoma**

Jianjun Xu,^†1^ Zhe Su,^†1^ Xiang Cheng,^†2^ Shaobo Hu, ^†1^ Wenjie Wang,^1^ Tianhao Zou,^1^ Xing Zhou,^1^ Zifang Song,^1^ Yun Xia,^*3^ Yang Gao,^*1^ and Qichang Zheng^*1^

^1^Department of Hepatobiliary Surgery, Union Hospital, Tongji Medical College,

Huazhong University of Science and Technology, Wuhan 430022, China

^2^Cancer Center, Union Hospital, Tongji Medical College, Huazhong University of Science and Technology, Wuhan 430022, China.

^3^Department of General Surgery, Tongji Hospital, Tongji Medical College, Huazhong University of Science and Technology, Wuhan 430030, China.

^†^These authors have contributed equally to this work.

^*^Corresponding author: [Xiayun7373@126.com](mailto:Xiayun7373@126.com) (Yun Xia); [hzkjdgy@163.com](mailto:hzkjdgy@163.com) (Yang Gao); qc_zheng@hust.edu.cn (Qichang Zheng)

**Additional Figures**


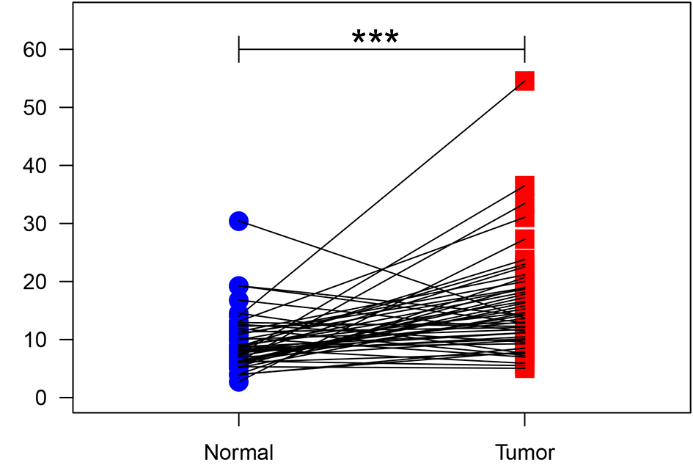


**Figure S1.** TCGA database-based comparison of PPT1 mRNA expression in the HCC tissues (n = 50) and its paired-normal tissues. ****P* <0.001.


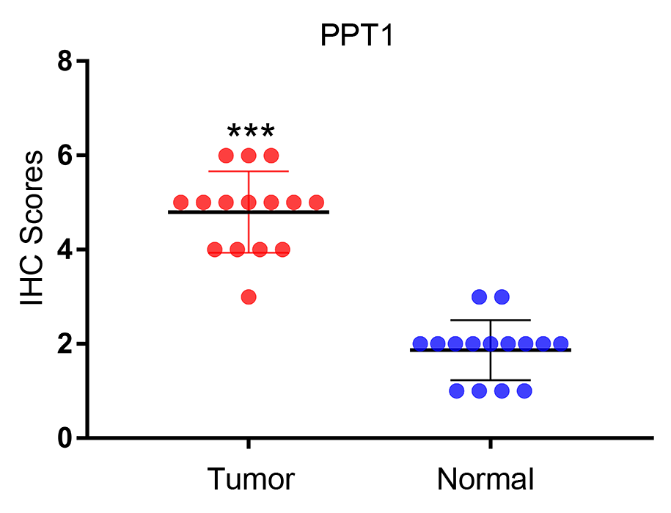


**Figure S2.** Comparison of PPT1 protein expression in patient-derived HCC tissues (n = 15) and its paired-normal tissues. ****P* <0.001.


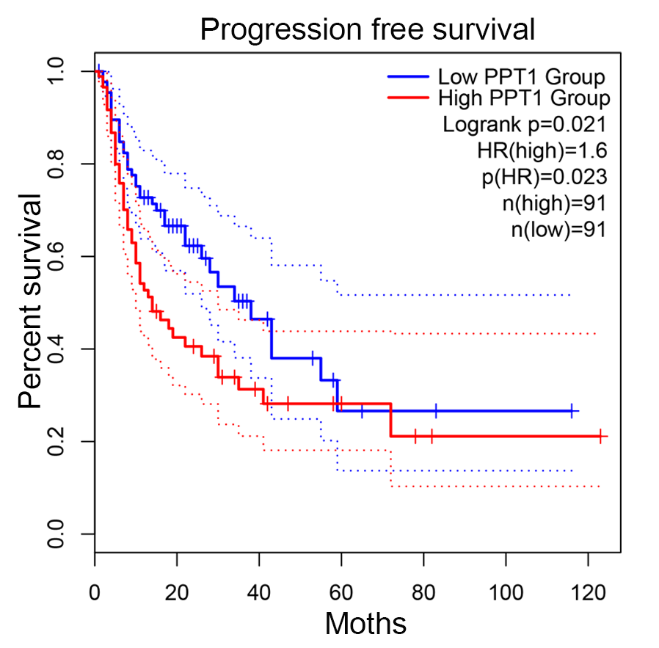


**Figure S3.** Kaplan–Meier curves of progression free survival (PFS). High PPT1 expression is correlated with poor PFS in HCC patients.


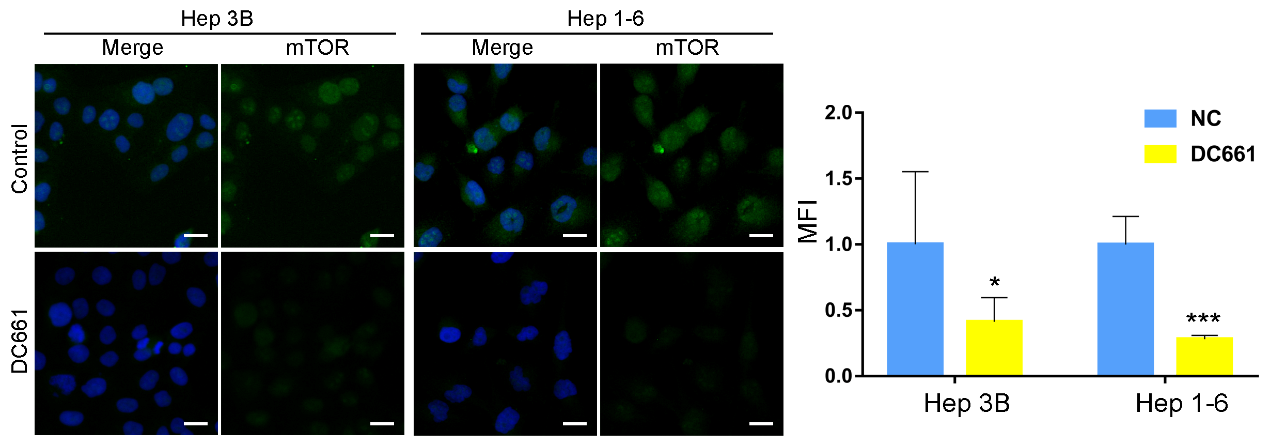


**Figure S4.** Immunofluorescence staining and semiquantitative analysis of mTOR in Hep 3B and Hep 1-6 cells treated with DC661 (3 μM, 6 h). Scale bar, 20 μm. Data represent mean ± SD; **P* < 0.05; ****P* < 0.001, compared with control group.


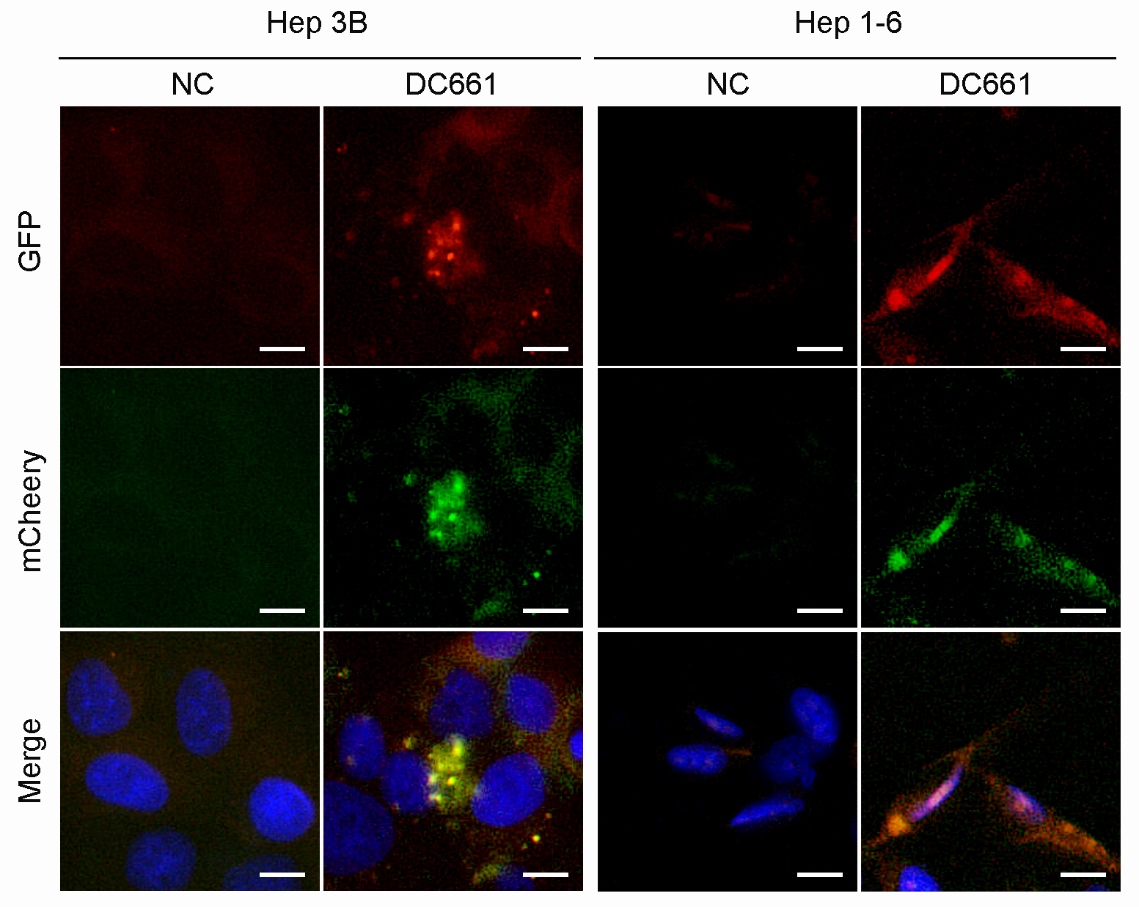


**Figure S5.** Photographs of fluorescence microscopy of punctate fluorescence of a transfected mCherry-GFP-LC3 construct in Hep 3B and Hep 1-6 cells after treatment with DC66 (3 μM, 6 h), Hoechst 33258 labels the nucleus. Scale bar, 10 μm.


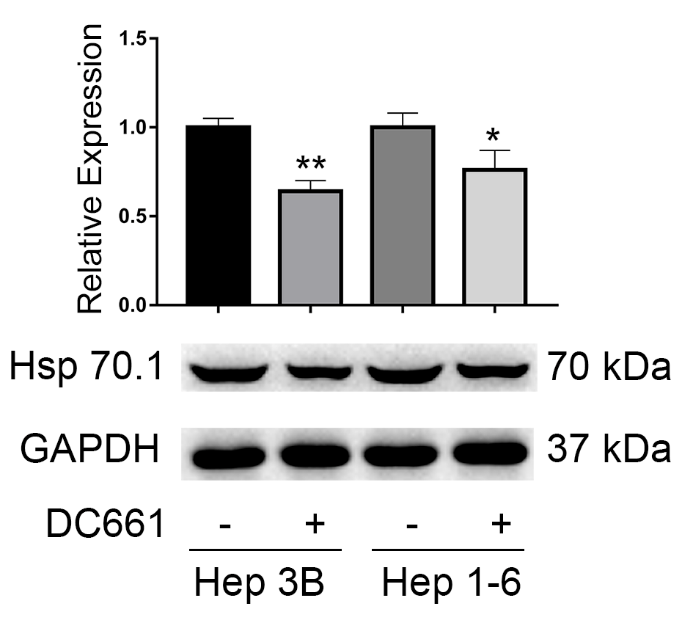


**Figure S6.** Western blot showing HSP70.1 decrease in Hep 3B and Hep 1-6 cells treated with DC661 (3 μM, 6 h). Data represent mean ± SD; **P* < 0.05; ***P* < 0.01, compared with control group.


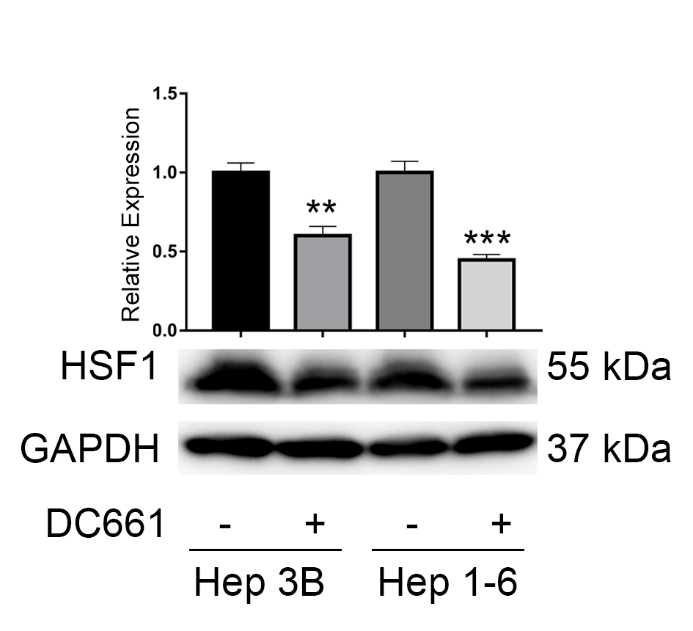


**Figure S7.** Western blot showing HSF1 decrease in Hep 3B and Hep 1-6 cells treated with DC661 (3 μM, 6 h). Data represent mean ± SD; ***P* < 0.01; ****P* < 0.001, compared with control group.


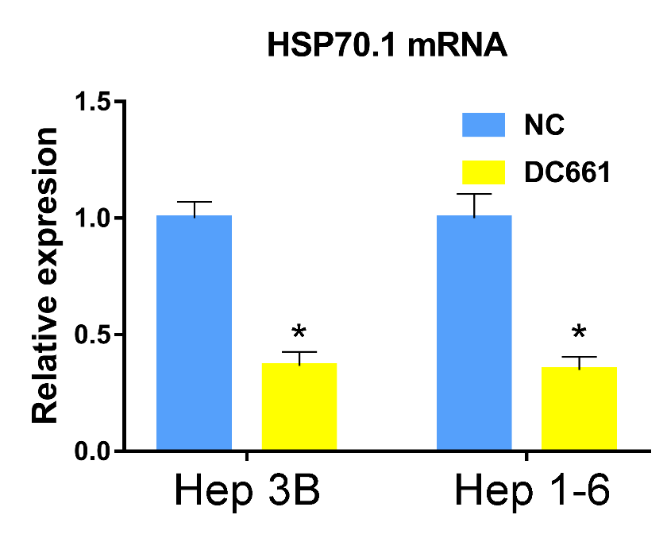


**Figure S8.** The expression of HSP70.1 mRNA in Hep 3B and Hep 1-6 cells treated with DC661 (3 μM, 6 h). Data represent mean ± SD; **P* < 0.05, compared with control group.


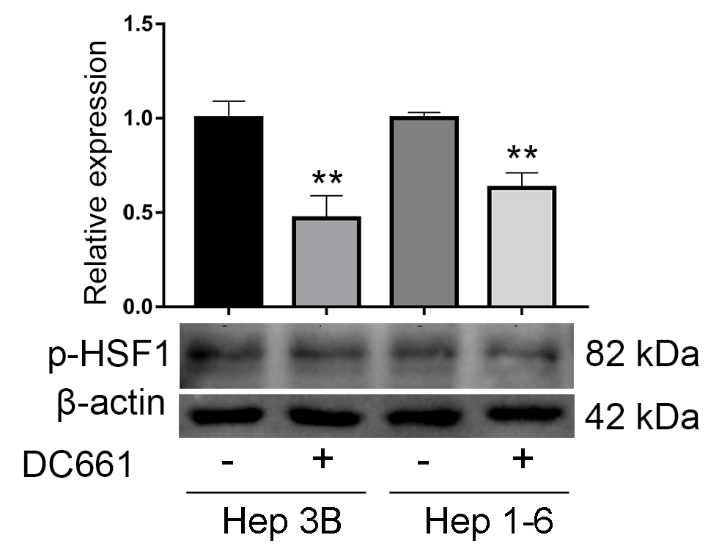


**Figure S9.** Western blot showing p-HSF1(ser 326) decrease in Hep 3B and Hep 1-6 cells treated with DC661 (3 μM, 6 h). Data represent mean ± SD; ***P* < 0.01, compared with control group.


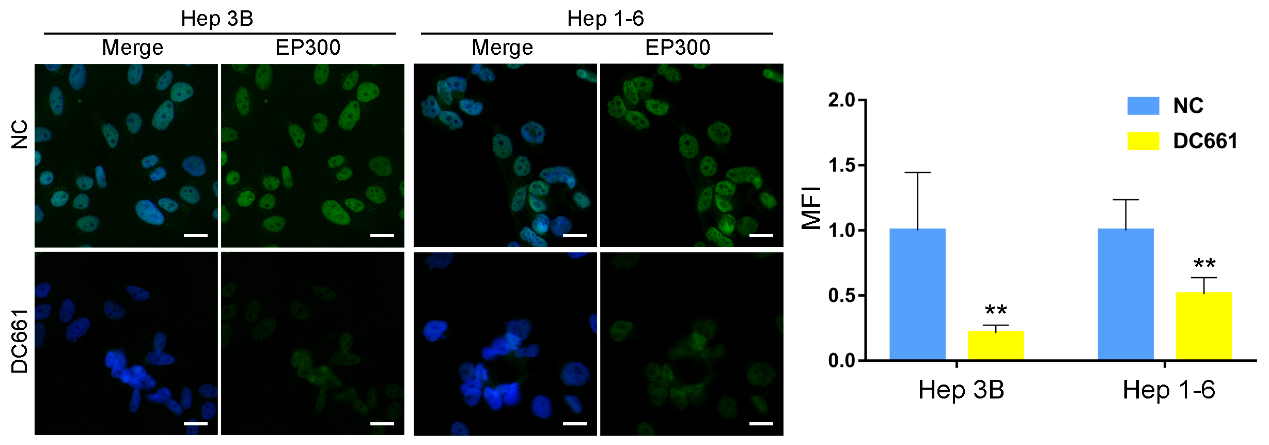


**Figure S10.** Immunofluorescence staining and semiquantitative analysis of EP300 in HCC cells after treatment with DC661 (3 μM, 6 h). Scale bar, 20 μm. Data represent mean ± SD; ***P* < 0.01, compared with control group.


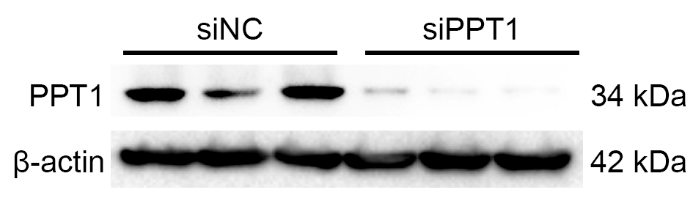


**Figure S11.** Western blot analysis of PPT1 in siNC and siPPT1 Hep 3B cells.


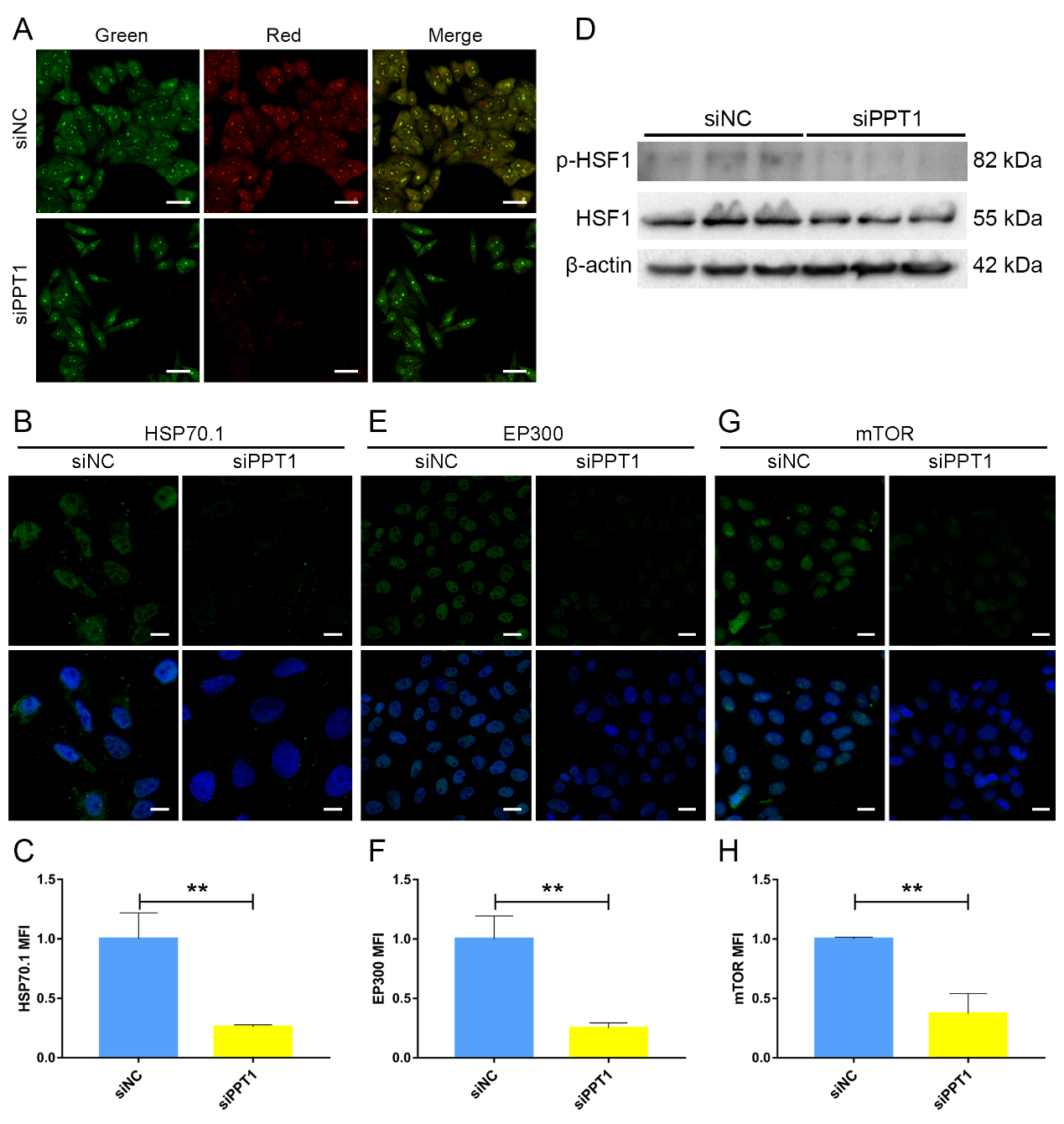


**Figure S12.** The effect of PPT1 knockdown on lysosomal membrane permeability and signal pathway. (A) Fluorescence images of AO staining in siNC and siPPT1 Hep 3B cells. Scale bar, 50 μm. (B,C) Immunofluorescent staining and semiquantitative analysis of HSP70.1 in siNC and siPPT1 Hep 3B cells. Scale bar, 10 μm. Data represent mean ± SD; ***P* < 0.01. (D) Western blot analysis of HSF1 and p-HSF1 in siNC and siPPT1 Hep 3B cells. (E,F) Immunofluorescent staining and semiquantitative analysis of EP300 in siNC and siPPT1 Hep 3B cells. Scale bar, 20 μm. Data represent mean ± SD; ***P* < 0.01. (G,H) Immunofluorescent staining and semiquantitative analysis of mTOR in siNC and siPPT1 Hep 3B cells. Scale bar, 20 μm. Data represent mean ± SD; ***P* < 0.01.


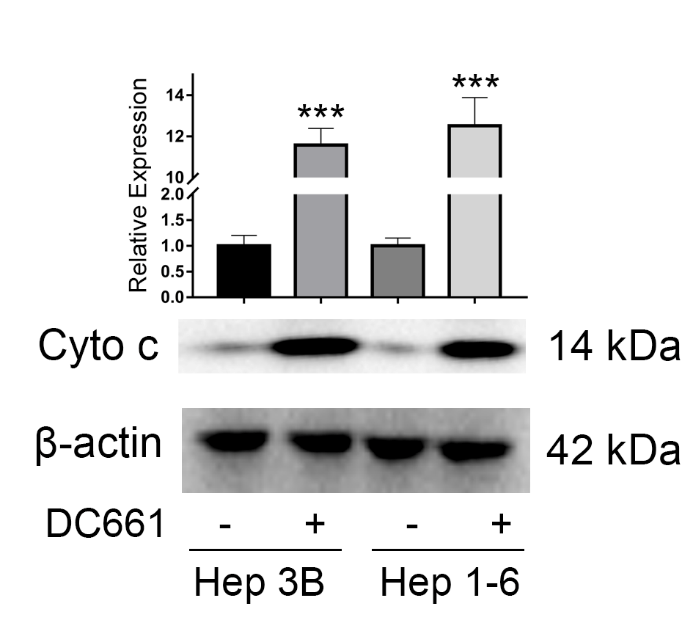


**Figure S13.** Western blot analysis of cytochrome c release in Hep 3B and Hep 1-6 cells treated with DC661 (3 μM, 6 h). Data represent mean ± SD; ****P* < 0.001, compared with control group.


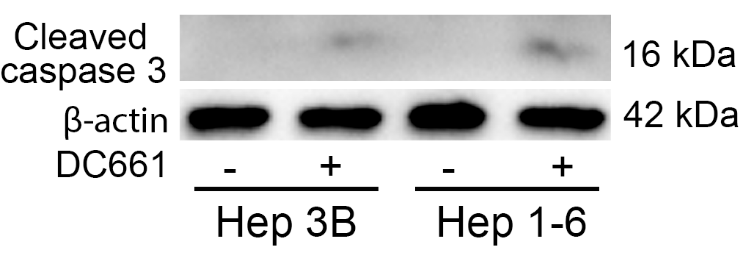


**Figure S14.** Western blot analysis of caspase-3 activation in Hep 3B and Hep 1-6 cells treated with DC661 (3 μM, 6 h).


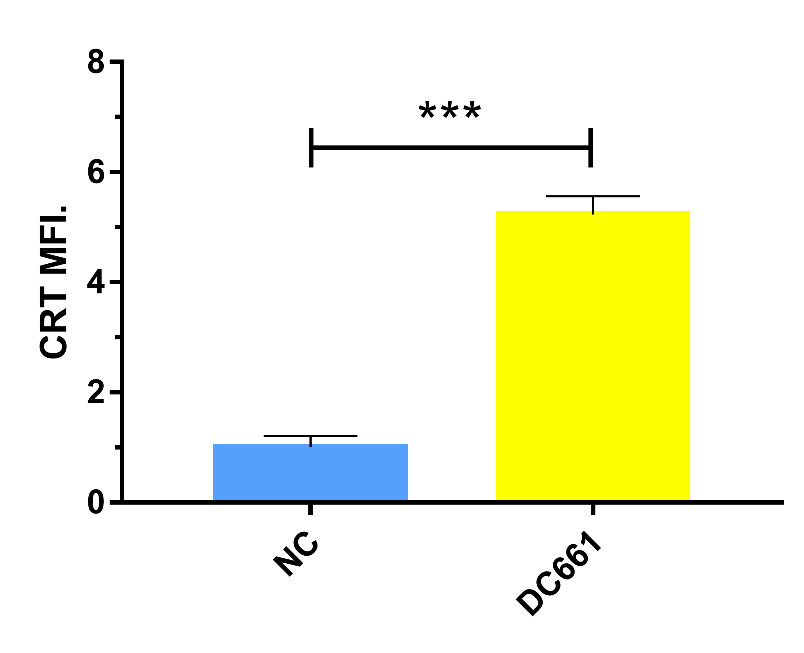


**Figure S15.** Flow cytometric analyses of CRT expression on cell membrane in Hep 3B and Hep 1-6 cells treated with DC661 (3 μM, 6 h). Data represent mean ± SD; ****P* < 0.001, compared with control group.


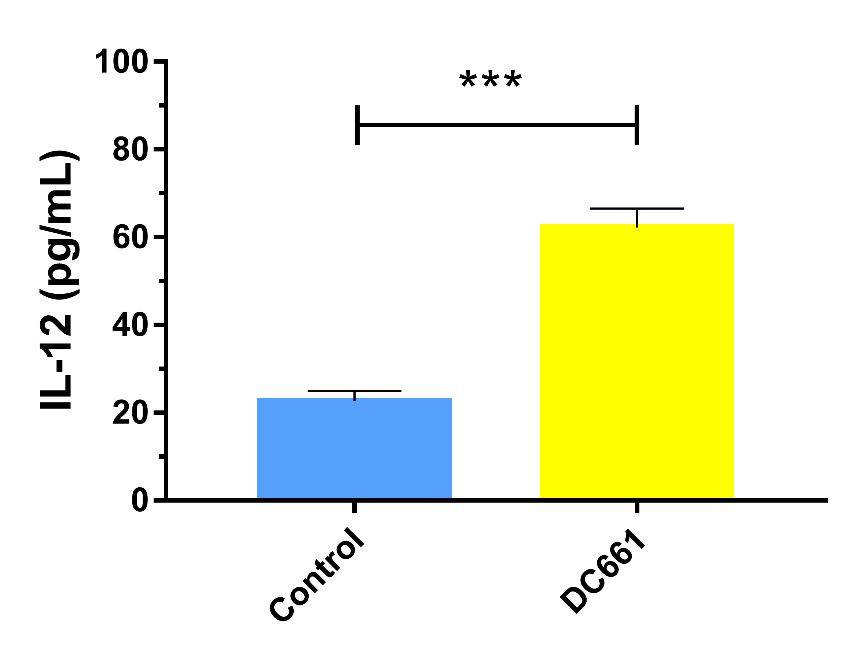


**Figure S16.** IL-12 levels in the serum from tumor-bearing mice after different treatments as measured by Elisa kit. Data represent mean ± SD; ****P* < 0.001, compared with control group.


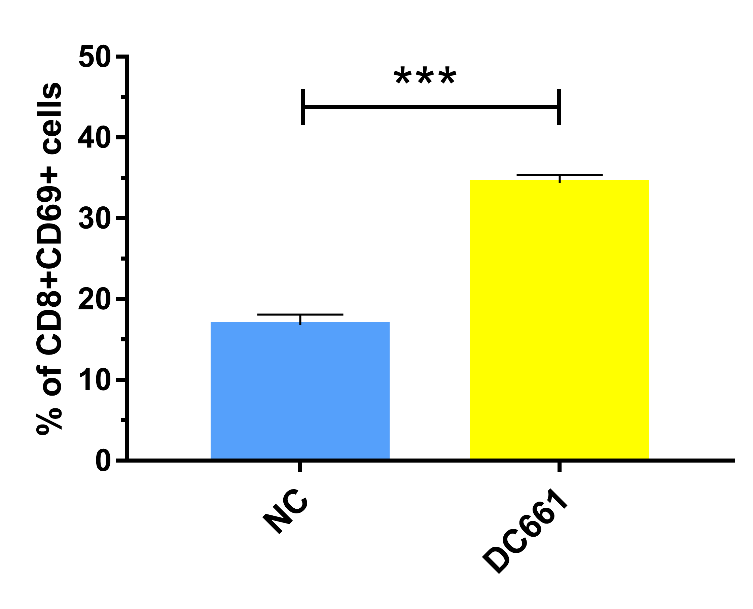


**Figure S17.** Flow cytometric analyses of activated CD8^+^ T cells (CD8^+^CD69^+^) from the tumor microenvironment of DC661- or vehicle-treated Hep 1-6 tumor-bearing mice. Data represent mean ± SD; ****P* < 0.001, compared with control group.


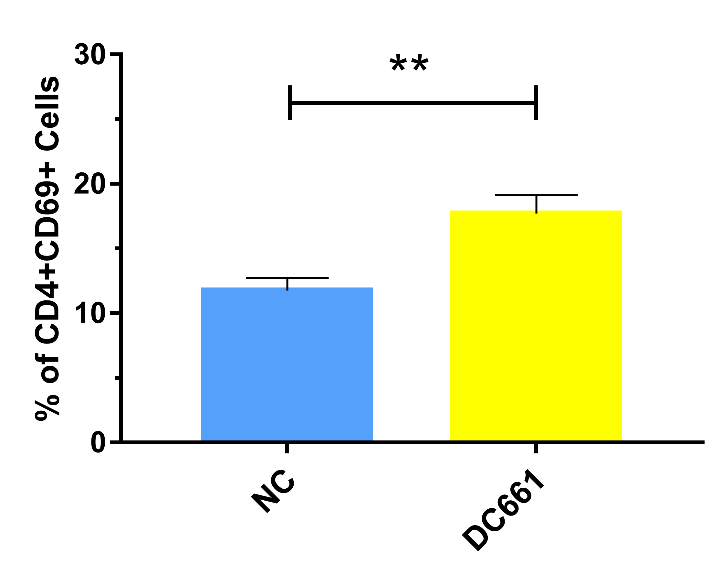


**Figure S18.** Flow cytometric analyses of activated CD4^+^ T cells (CD4^+^CD69^+^) from the tumor microenvironment of DC661- or vehicle-treated Hep 1-6 tumor-bearing mice. Data represent mean ± SD; ***P* < 0.01, compared with control group.


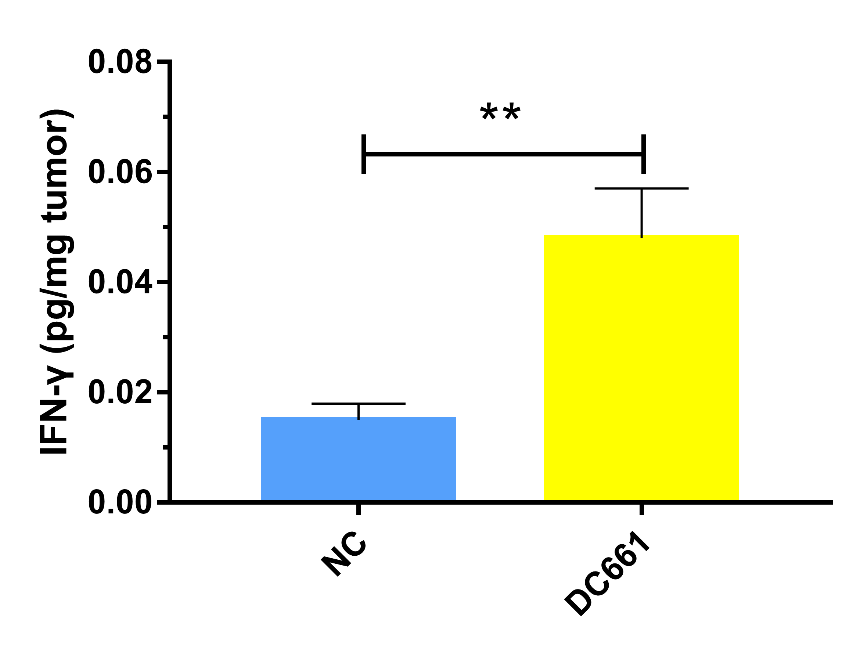


**Figure S19.** Expression of IFN-γ in tumor microenvironment was detected by Elisa. Data represent mean ± SD; ***P* < 0.01, compared with control group.


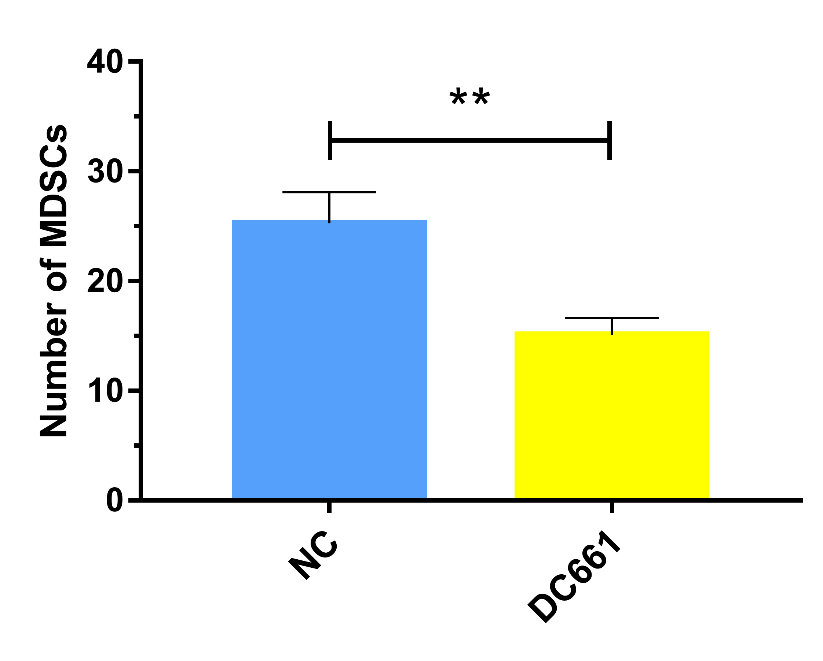


**Figure S20.** Flow cytometric analyses of MDSCs (CD45^+^CD11b^+^Gr-1^+^) in tumor. Data represent mean ± SD; ***P* < 0.01, compared with control group. Myeloid-derived suppressor cells: MDSCs.


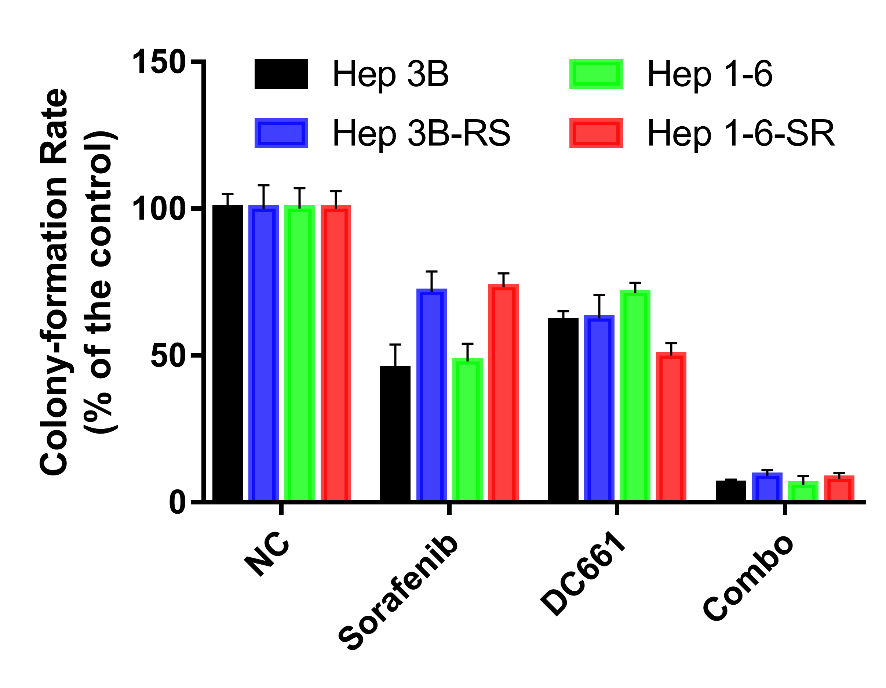


**Figure S21.** Quantification for the colony formation assay after different treatments.


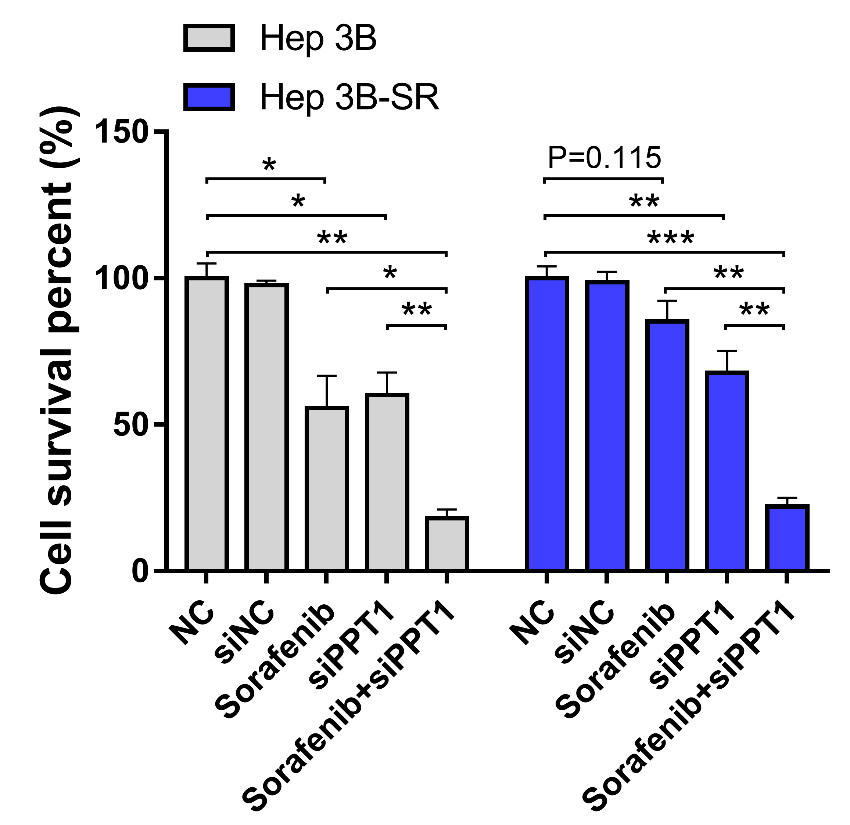


**Figure S22.** Cell viability in untreated or siNC and siPPT1 HCC cells after treatment with sorafenib (1.55 μM, 48 h) was determined by CCK-8 assay. **P* < 0.05; ***P* < 0.01; ****P* < 0.001.


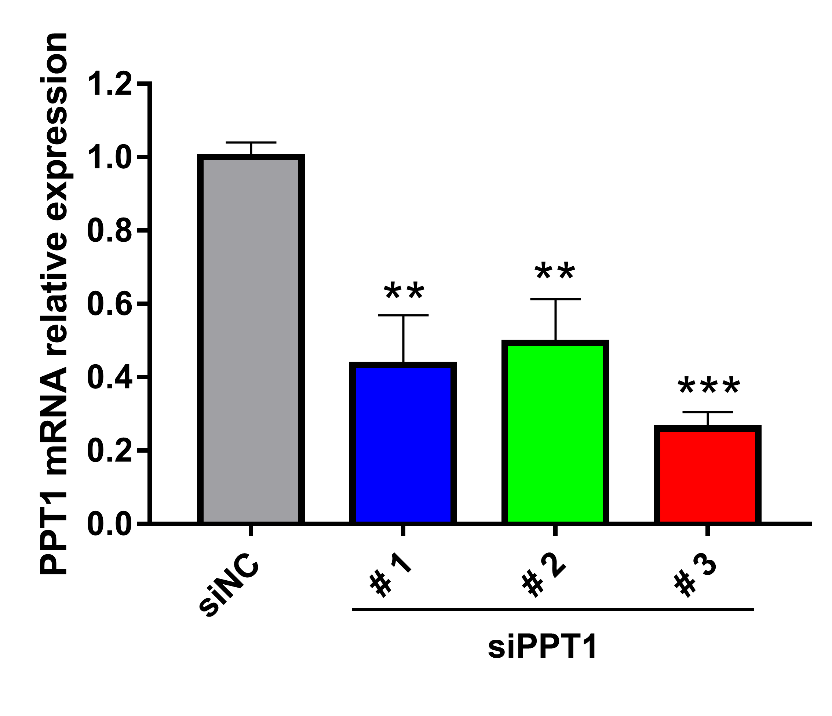


**Figure S23.** The expression of PPT1 mRNA after transfected with siPPT1. Data represent mean ± SD; ***P* < 0.01, ****P* < 0.001, compared with siNC group.

**Additional Table**

**Table S1.** The sequences for siPPT1.

| siRNA | sense/anti-sense | sequences |
| --- | --- | --- |
| siPPT1 #1 | sense | 5’-CCACAUCAUACCAUUCCUUTT-3’ |
|  | anti-sense | 5’-AAGGAAUGGUAUGAUGUGGTT-3’ |
| siPPT1 #2 | sense | 5’-GCUCUCACAUCUGUGACUUTT-3’ |
|  | anti-sense | 5’-AAGUCACAGAUGUGAGAGCTT-3’ |
| siPPT1 #3 | sense | 5’-GCAUCUUCUUGGCAGAUAUTT-3’ |
|  | anti-sense | 5’-AUAUCUGCCAAGAAGAUGCTT-3’ |
| siNC | sense | 5’-UUCUCCGAACGUGUCACGUTT-3’ |
|  | anti-sense | 5’-ACGUGACACGUUCGGAGAATT-3’ |
